# Genetic architecture of complex agronomic traits examined in two testcross populations of rye (*Secale cereale* L.)

**DOI:** 10.1186/1471-2164-13-706

**Published:** 2012-12-17

**Authors:** Thomas Miedaner, Marlen Hübner, Viktor Korzun, Brigitta Schmiedchen, Eva Bauer, Grit Haseneyer, Peer Wilde, Jochen C Reif

**Affiliations:** 1 State Plant Breeding Institute, Universität Hohenheim, Stuttgart, 70593, Germany; 2 KWS LOCHOW GmbH, Bergen, 29303, Germany; 3 Plant Breeding, Technische Universität München, Freising, 85354, Germany

## Abstract

**Background:**

Rye is an important European crop used for food, feed, and bioenergy. Several quality and yield-related traits are of agronomic relevance for rye breeding programs. Profound knowledge of the genetic architecture of these traits is needed to successfully implement marker-assisted selection programs. Nevertheless, little is known on quantitative loci underlying important agronomic traits in rye.

**Results:**

We used 440 F_3:4_ inbred lines from two biparental populations (Pop-A, Pop-B) fingerprinted with about 800 to 900 SNP, SSR and/or DArT markers and outcrossed them to a tester for phenotyping. The resulting hybrids and their parents were evaluated for grain yield, single-ear weight, test weight, plant height, thousand-kernel weight, falling number, protein, starch, soluble and total pentosan contents in up to ten environments in Central Europe. The quality of the phenotypic data was high reflected by moderate to high heritability estimates. QTL analyses revealed a total of 31 QTL for Pop-A and 52 for Pop-B. QTL x environment interactions were significant (P < 0.01) in most cases but variance of QTL main effect was more prominent.

**Conclusions:**

QTL mapping was successfully applied based on two segregating rye populations. QTL underlying grain yield and several quality traits had small effects. In contrast, thousand-kernel weight, test weight, falling number and starch content were affected by several major QTL with a high frequency of occurrence in cross validation. These QTL explaining a large proportion of the genotypic variance can be exploited in marker-assisted selection programs and are candidates for further genetic dissection.

## Background

Rye (*Secale cereale* L.) is an important European crop grown on 4 million hectares. Main producers are Poland, Germany, and the Russian Federation sharing about 60% of the world production in 2010 [1]. Rye grain is used for bread making, as feed for livestock, for ethanol production, and as substrate in biogas plants. Hybrid cultivars based on the high yielding heterotic pattern Petkus times Carsten [2] were successfully introduced in Germany three decades ago. Hybrids are now grown on about 60-70% of the total rye acreage owing to their yield superiority and better uniformity as compared to open-pollinating varieties. German commercial hybrid varieties are also released and grown in Denmark, Austria, and Poland.

The economically most important trait in hybrid rye breeding is grain yield. Other agronomic relevant traits are lodging resistance, plant height, thousand-kernel weight (TKW), and falling number as indirect trait for pre-harvest sprouting resistance. Relevant quality traits differ depending on the end use of rye. For baking quality, for example, high pentosan and starch content play a major role coupled with a low protein content. In contrast, for feeding purposes, protein content should be maximized and pentosan content minimized. For ethanol production, breeding for high starch content is of central importance. Several pairs of the above mentioned quality traits are negatively correlated such as protein and starch content, which hampers simultaneous breeding for the different end uses of rye.

The availability of reliable diagnostic markers for large effect QTL underlying the above mentioned traits, would tremendously alleviate breeding rye varieties meeting these often contradictory demands. Unfortunately, not much is known on the genetic architecture of most of these traits and diagnostic markers are not available. The main cause is that rye was lagging far behind other crops in terms of genomic resources. Until recently, only about 250 (simple sequence repeat, SSR) markers were available [3,4]. This changed with the development of DArT (diversity array technique) markers [5,6] and, more recently, a 5 k-SNP (single nucleotide polymorphism) array [7]. Until now, only a few QTL mapping surveys for a few specific traits were published in rye studying the pleiotropically acting reduced height gene locus *Ddw1*[8], *in vitro* response [9], and α-amylase activity and related traits, e.g. [10,11]. Genomic segments responsible for agronomic traits were detected in two introgression libraries derived from an Iranian primitive rye [12,13] using SSR and amplified fragment length polymorphism (AFLP) markers.

We report the first genome-wide QTL analysis across two segregating rye populations for a comprehensive set of agronomic traits. We successfully identified QTL responsible for the expression of each of five yield- and quality related traits (grain yield, plant height, TKW, single-ear weight, test weight, falling number, total and soluble pentosan, starch, and protein contents) and analyzed their reproducibility across environments. We analyzed trait associations at the overall level but also with regard to their underlying QTL and report QTL with major effects useful for marker-assisted selection and map-based cloning.

## Methods

### Plant material

Three parental winter rye elite inbred lines (Lo90-N, Lo115-N, Lo117-N) were chosen for this study. Two segregating populations, each consisting of 220 F_2_ lines were generated by crossing (1) inbred line Lo115-N with inbred line Lo90-N (Pop-A) and (2) inbred line Lo115-N with inbred line Lo117-N (Pop-B). Parents belong to the Petkus gene pool (seed parent) and possessed normal cytoplasm (N). F_2_ plants were randomly forwarded to F_3_ generation by single-seed descent and tested in F_4_ generation (named F_3:4_ lines in the following) The 440 randomly taken F_3:4_ lines were crossed to an unrelated cytoplasmic-male sterile (CMS) single-cross tester of the Petkus gene pool (seed parent pool) by open pollination between isolation walls. The resulting three-way crosses of the type (A · B) × F_3:4_ line will be addressed as testcrosses throughout the paper. They consisted only of non-restorer materials (Petkus × Petkus), which required the supply of external pollen for fertilization during field trials. For this, a 1:1-mixture of the two pollen-shedding open-pollinated varieties ‘Danko’ and ‘Recrut’ was planted in the alleys and in stripes in a regularly distance of ten plots through the whole experiment. These two population cultivars were chosen to result in an extended flowering period. All plant materials used in this study (except ‘Danko’) were kindly supplied by KWS LOCHOW GMBH, Bergen, Germany.

### Field experiments and traits

Field experiments were conducted in the years 2010 and 2011 at five locations: (1) Wohlde (WOH), Germany, N52.8°, E10.0°, 80 m above sea level; (2) Beckedorf (BEK), Germany, N52.5°, E10.3°, 80 m above sea level; (3) Petkus (PET), Germany, N51.6°, E13.2°, 130 m above sea level; (4) Stuttgart/Hohenheim (HOH), N48.4°, E9.1°, 400 m above sea level, and (5) Walewice (WAL), Poland, N52.6°, E19.4°, 184 m above sea level. The location × year combinations were referred to as environments in the following.

Both populations with each of 220 lines were evaluated in field trials together with their parents (repeated 9 times) and arranged in an incomplete 24 × 10 lattice design with two replications. Plot size ranged from 5 to 6 m^2^ and seeding rate varied from 150–200 kernels m^-2^. Standard production practices of mineral fertilizer, herbicides and fungicides applications were used following local standards. To avoid lodging, growth regulators were applied three times. Caused by insufficient rainfall during both growing seasons irrigation was applied at WOH, PET, and WAL by a drip irrigation system. Five drip lines per plot were established to ensure a consistent irrigation in each plot depending on the particular local condition. Data were recorded for plant height (cm), grain yield (Mg ha^-1^), 1000-kernel weight (TKW, g), test weight (kg), single ear-weight (g), falling number (sec.), total pentosan (%), soluble pentosan (%), protein (%), and starch contents (%). For the first five traits data across ten environments and for the latter five traits data across six environments (WOH, PET, WAL in 2010 and 2011) were available. For falling number, data from one environment (WAL10) was missing. Plots were machine-planted and combine-harvested and grain yield was adjusted to a moisture concentration of 140 g H_2_O kg^-1^. The traits total pentosan (%), soluble pentosan (%), protein (%), and starch contents (%) were determined by near-infrared reflectance spectroscopy (NIRS, calibration by KWS SAAT AG, Einbeck) taking two samples per plot. Test weight refers to the weight of 100 l of grain.

### Phenotypic data analysis

We used a two-step procedure to analyse the phenotypic data. In a first step, ordinary lattice analyses of variance were performed for each environment and population separately [14]. In a second step, adjusted means of each genotype were used to estimate variance components across environments for each of the two segregating populations [15]. We assumed in this model that genotype and environment effects were random. Heritability on an entry-mean basis was estimated from the variance components as the ratio of genotypic to phenotypic variance [16]:

(1) $$h^{2}=\;\frac{\sigma_{G}^{2}}{\sigma_{G}^{2}+\frac{\sigma_{\mathrm{GxE}}^{2}}{E}+\frac{\sigma_{e}^{2}}{\mathrm{ER}}},$$

where σ^2^_G_ denotes the genotypic variance, σ^2^_GxE_ the genotype × environment interaction variance, and σ^2^_e_ the error variance, E and R are the numbers of environments and replications, respectively. Test of skewness and kurtosis was performed for each trait [15], pp. 79–81]. All statistical analyses were performed with the PLABSTAT software package [17].

### Genetic linkage map construction

Genomic DNA was extracted from leaf samples of the two segregating populations at seedling stage using the procedure described by [18]. Pop-A (219 lines) was genotyped by simple sequence repeat (SSR) and single nucleotide polymorphism (SNP) markers, Pop-B (202 lines) by SSR- and diversity array technology (DArT)-markers, because SNP markers were not available. For SSR genotyping we followed established procedures [3]. Briefly, PCR products were separated on an ABI3130×l Genetic Analyzer (Applied Biosystems) according to manufacturer’s instructions. Allele identity was assigned using the GENEMAPPER software (Applied Biosystems). The Illumina iSelect Rye5k SNP array [7] was used for genotyping Pop-A. Briefly, a total of 200 ηg genomic DNA per plant was used for SNP genotyping on Illumina’s iScan platform using the Infineon HD assay for Pop-A and the GoldenGate assay for Pop-B following the manufacturer’s protocol. Raw hybridization intensity data processing, clustering, and genotype calling were performed using the genotyping module in the BeadStudio package (Illumina, San Diego, CA, USA). Data of the Rye5k SNP array for Pop-A were generated by Eva Bauer’s group at the Technische Universität München, Freising, Germany. SSR genotyping for both populations was done by KWS LOCHOW GMBH. DArT genotyping with the current rye array was performed by Triticarte Pty Ltd, Yarralumla, ACT, Australia (http://www.triticarte.com.au).

For the construction of the genetic linkage maps, polymorphic markers in each population were transformed into genotype codes according to the parental score. For quality checks a pre-selection with regard to their deviation from expected segregation ratio was performed. The genetic linkage maps were constructed with the software JoinMap® 4.0 [19]. Seven linkage groups were established and chromosome names and orientation were assigned to linkage groups based on a subset of markers for which the positions have been published previously [5]. Locus order and genetic distances in centimorgan (cM) along the chromosome were calculated with the maximum likelihood algorithm using Kosambi’s mapping function [20]. After each run post mapping quality tools provided by JoinMap 4.0 were used excluding markers which do not fit into the genetic linkage map. To estimate average marker densities, markers with the same position were considered as single marker (unique locus) to avoid an overestimation of the marker density.

### QTL analyses

For each trait and each population, QTL analyses were based on the genetic linkage map of each population and on adjusted entry means using the software PLABQTL [21]. Markers with a distance below 1 cM were excluded by the software. Our QTL study is based on testcross performance of F_3:4_ families. The genetic make-up of our populations allows only the detection of main effect QTL contributing to the additive genetic variation. Therefore, we assumed an additive model as outlined by [22]. QTL analyses were based on Composite Interval Mapping (CIM) with a multiple regression approach [23]. Cofactors were selected by stepwise regression according to Miller [24], pp. 49] with an “F-to-enter” and “F-to-delete” value of 3.5. Estimates of QTL positions were attained at the position where the LOD score assumed its maximum in the region under consideration. The critical LOD threshold was analysed empirically for each trait using 1,000 permutation runs [25]. The proportion of the phenotypic variance explained by the QTL was determined by the estimator *R*^*2*^_*adj*_ as described by [22]. Additionally, five-fold cross validation (CV) was applied to determine the bias of *R*^2^ explained by detected QTL resulting in *R*^*2*^_*CV*_. For this, the entire data set (DS) was split into five genotypic subsamples. Means from four out of five subsamples served as estimation set (ES) for QTL detection, localization, and estimation of genetic effects. The remaining subset forms the test set (TS) in which predictions derived from the ES are tested for their validity by correlating predicted and observed data. By permuting the respective subsets used for the ES and TS, five different cross-validation runs were used [22]. As a measure of reliability we give for each QTL the frequency of occurrence, i.e. the percentage of runs in which the QTL was detected within 1,000 cross-validation runs. The proportion of genotypic variance (*p*_*G*_) explained by the model was calculated: *p*_*G*_ = *R*^*2*^_*adj*_/*h*^*2*^, where *R*^*2*^_*adj*_ is the adjusted proportion of phenotypic variance explained by the model and h^2^ is the heritability of the trait [26].

## Results

### Phenotyping revealed a large genetic variation for the ten agronomic and quality traits

**Figure 1:**
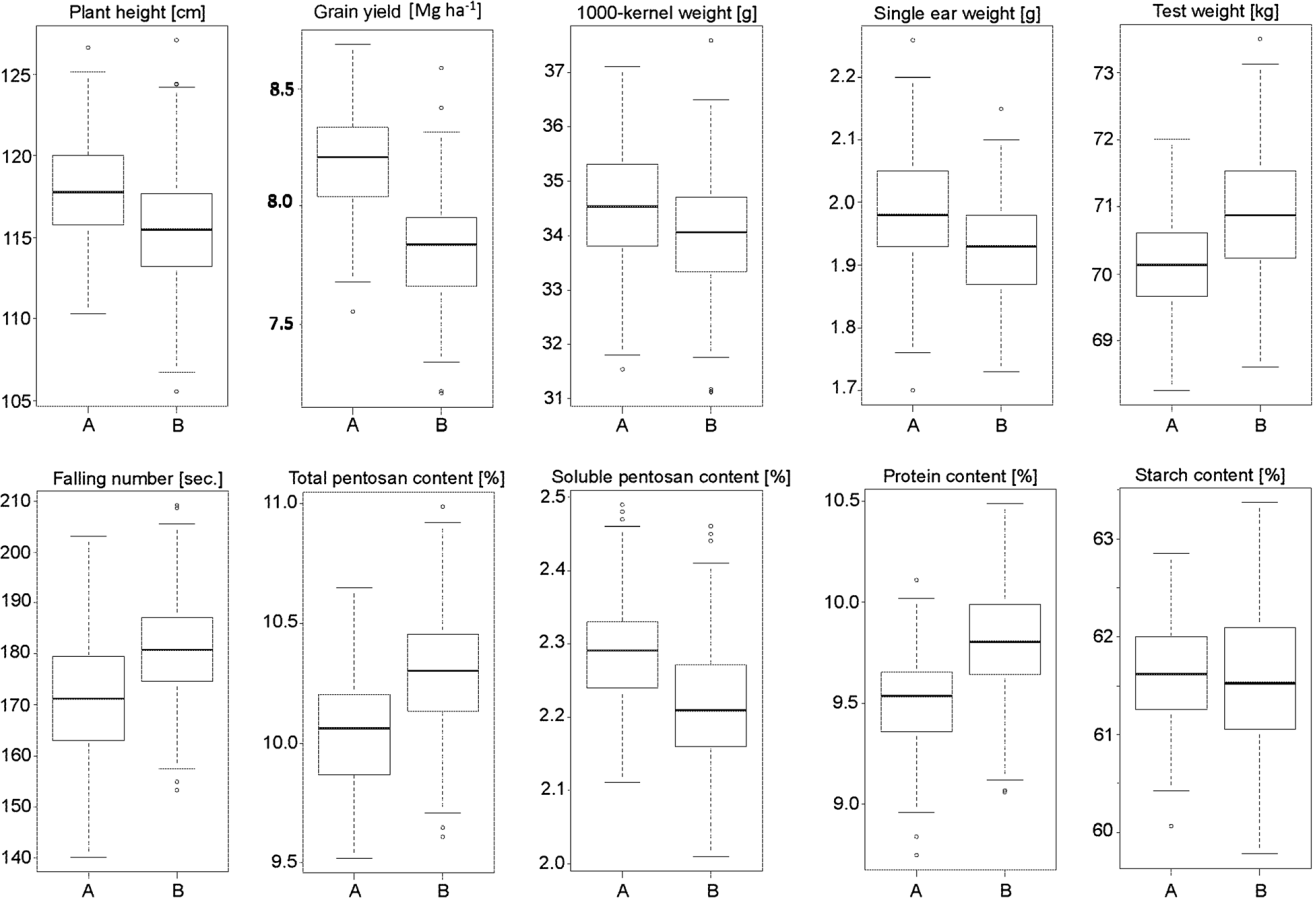
**Box-Whisker plots for agronomic and quality traits.** Data based on each of 220 testcross progenies of two populations (**A**, **B**) across ten (plant height and yield-related traits) and six (quality traits) environments.

For all traits significant (P < 0.01) genotypic variation was observed in both populations (Table 1, Additional file 1). Variances due to genotype × environment interaction effects were also significantly (P < 0.05) greater than zero with the exception of starch content. Intensive phenotyping at up to ten environments led to moderate to high heritability estimates with the exception of falling number and soluble pentosan content that had lower estimates (0.3-0.4). Although parents of Pop-A only slightly differed in their testcross performance, the segregation variance of their progenies was similar to that of Pop-B. In contrast, parents varied widely for Pop-B: Lo117 was lower yielding, shorter and had a lower TKW than Lo115. Progeny means resembled parental means well. Both populations differed in their medians for all traits, but the genotypic variances indicated by the 50% quantile were rather similar (Figure 1). All phenotypic data did not significantly deviate from normal distribution.

**Table 1:** First and second degree statistics of rye testcross progenies

**Traits**	**Parental mean**		**Testcross progenies**				
	**1**	**2**	**Mean**	***σ***_***G***_^**2**^	***σ***_***GxE***_^**2**^	***σ***_***e***_^**2**^	**h**^**2**^
**Pop-A**							
Plant height [cm]	118.6	118.0	117.9	7.5**	3.4**	8.3	0.87
Yield-related traits:							
Grain yield [Mg ha^-1^]	8.33	7.95	8.18	0.035**	0.074**	0.077	0.70
TKW [g]	35.3	34.5	34.6	1.0**	0.9**	0.9	0.85
Single ear weight [g]	2.01	2.06	1.98	0.01**	0.01**	0.01	0.63
Quality-related traits:							
Test weight [kg]	69.7	70.0	70.2	0.45**	0.4**	0.2	0.88
Falling number [s] ^a^	173.3	174.0	171.8	62.2**	124.9**	345.1	0.44
Total pentosan content [%]	10.10	9.89	10.04	0.04**	0.08**	0.04	0.65
Soluble pentosan content [%]	2.29	2.25	2.29	0.002**	0.01**	0.01	0.33
Protein content [%]	9.55	9.53	9.51	0.02**	0.09**	0.06	0.48
Starch content [%]	61.53	61.61	61.62	0.23**	0.23	0.17	0.77
**Pop-B**							
Plant height [cm]	117.4	112.4	115.6	12.0**	4.5**	6.3	0.92
Yield-related traits:							
Grain yield [Mg ha^-1^]	8.44	7.33	7.82	0.037**	0.087**	0.071	0.70
TKW [g]	35.1	33.8	34.0	0.9**	0.9**	0.8	0.85
Single ear weight [g]	1.93	1.94	1.92	0.003**	0.005**	0.01	0.52
Quality-related traits:							
Test weight [kg]	69.9	71.8	70.9	0.8**	0.4**	0.3	0.92
Falling number [s] ^a^	185.6	175.2	180.7	35.8**	41.0*	302.9	0.39
Total pentosan content [%]	10.32	10.35	10.30	0.04**	0.09**	0.04	0.63
Soluble pentosan content [%]	2.33	2.18	2.22	0.003**	0.01**	0.01	0.46
Protein content [%]	9.70	10.0	9.81	0.06**	0.08**	0.05	0.74
Starch content [%]	61.40	61.45	61.53	0.44**	0.23	0.14	0.88

Means of parents (1 = Lo115, 2 = Lo90 in Pop-A and Lo117 in Pop-B, respectively) and each of 220 testcross progenies of two populations (Pop-A, Pop-B), estimates of variance components (genotype, *σ*_*G*_^2^; genotype x environment interaction, *σ*_*GxE*_^2^; error, *σ*_*e*_^2^), and entry-mean heritabilities (h^2^) for agronomic and quality traits evaluated across six to ten environments.

^a^Analysis across 5 environments.

Coefficients of phenotypic correlation among traits were low to moderate in both populations (Table 2). Significant (P < 0.01) correlations were found between single-ear weight and grain yield or TKW in Pop-A (r = 0.54; r =  0.51). Plant height was significantly (P < 0.01) correlated with TKW and starch content (r = 0.5; r = −/+0.3) in both populations and additionally with grain yield, test weight and single ear weight in Pop-A. Among the quality traits, consistent significant coefficients were found between starch content and test weight (r = 0.2 to 0.5) or protein content (r = −0.6 to −0.7).

**Table 2:** Coefficients of phenotypic correlation among ten traits

	**Plant**	**Yield-related traits**			**Quality-related traits**					
	**height**	**Grain yield**	**TKW**	**Single ear weight**	**Test weight**	**Falling number^a^**	**Total pentosan**	**Soluble pentosan**	**Protein**	**Starch**
Plant height	-	0.13	0.45**	0.08	−0.05	0.12	0.22**	0.25**	−0.02	0.31**
Yield-related traits:										
Grain yield	0.30**	-	0.13	0.36**	−0.10	0.16*	−0.11	−0.06	−0.47**	0.32**
TKW	0.46**	0.15*	-	0.02	−0.20	−0.09	0.33**	−0.33**	0.32**	−0.55**
Single ear weight	0.42**	0.54**	0.51**	-	−0.12	−0.05	−0.09	0.07	−0.33**	0.14*
Quality-related traits:										
Test weight	−0.25**	0.05	−0.13*	−0.27**	-	−0.06	−0.06	0.09	0.16*	0.23**
Falling number^a^	−0.14*	0.08	−0.25**	−0.13	0.03	-	0.04	−0.03	−0.23**	0.14*
Total pentosan content	0.08	0.00	0.05	−0.06	−0.30**	0.13*	-	−0.03	0.38**	−0.52**
Soluble pentosan content	−0.04	−0.02	−0.22**	−0.12	−0.32**	0.00	0.14*	-	−0.07	0.22**
Protein content	−0.02	−0.23**	−0.13	−0.23**	−0.11	0.03	0.10	0.22**	-	−0.74**
Starch content	−0.31**	0.12	−0.20**	−0.06	0.50**	0.05	−0.29**	−0.25**	−0.60**	-

Estimated for each of 220 testcross progenies of Pop-A (below diagonal) and Pop-B (above diagonal) across ten (agronomic traits) and six (quality traits) environments.

*,** Significant at the 0.05 and 0.01 probability level, respectively.

^a^Analysis across 5 environments.

### The joint use of SNP, DArT and SSR markers resulted in dense genetic linkage maps

**Figure 2:**
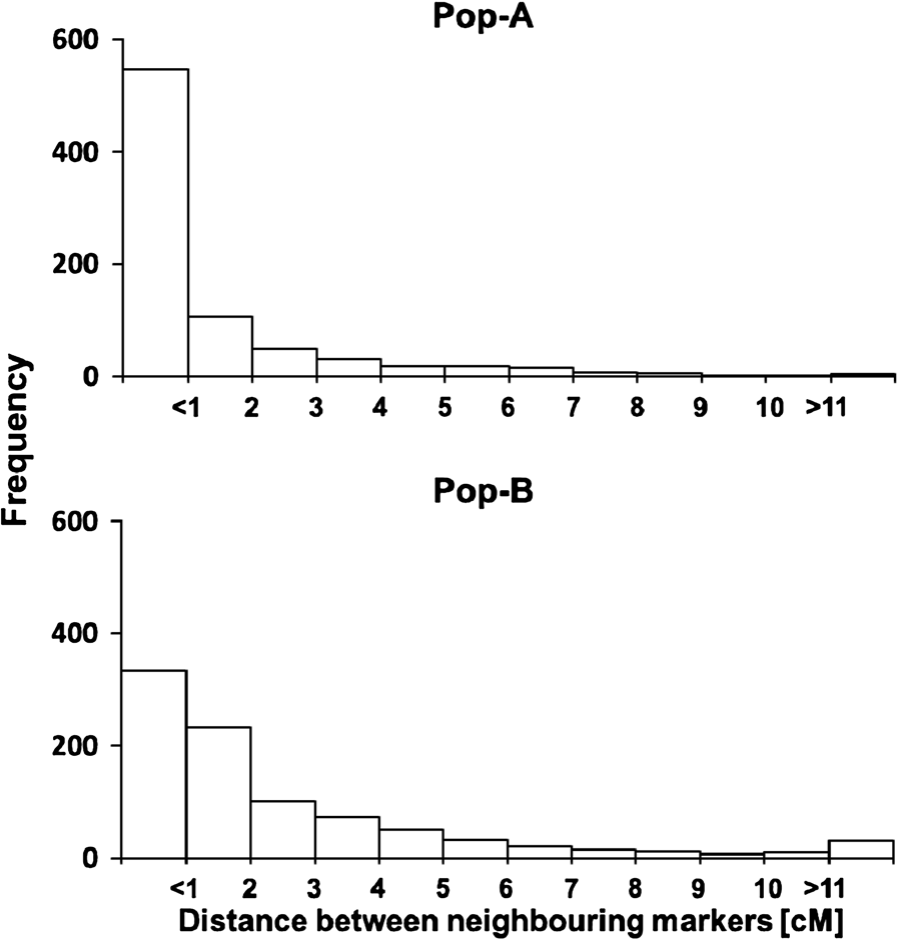
Frequency distributions of distances between neighbouring markers.

In this study, we used either a newly developed SNP marker array [7] or DArT markers for successfully generating high-density genetic linkage maps for both populations (Additional files 2 and 3). Finally, 813 and 921 markers were used for genetic linkage map construction for Pop-A and for Pop-B, respectively. The genetic linkage maps possess a total length of 980 cM for Pop-A and 2,349 cM for Pop-B. We found for Pop-A an average marker density of 1.48 cM and for Pop-B of 2.58 cM. The seven rye chromosomes were covered with each of 71 to 164 markers in Pop-A and 111 to 152 markers in Pop-B. The majority of neighbouring markers had a distance of less than 1 cM, only a few of the pairs had a genetic map distance larger than 10 cM (Figure 2). Map length and average marker density of the latter population are comparable with a rye population mapped by DArTs recently [5]. Obviously, the combination of DArT and SSR markers resulted in longer maps than that of SNP and SSR markers. However, for QTL mapping a marker distance of 15–20 cM is sufficient [27]. The maps, therefore, provide a solid basis for identifying genomic regions underlying the analyzed traits.

### Genomic regions controlling ten agronomic and quality traits

**Figure 3:**
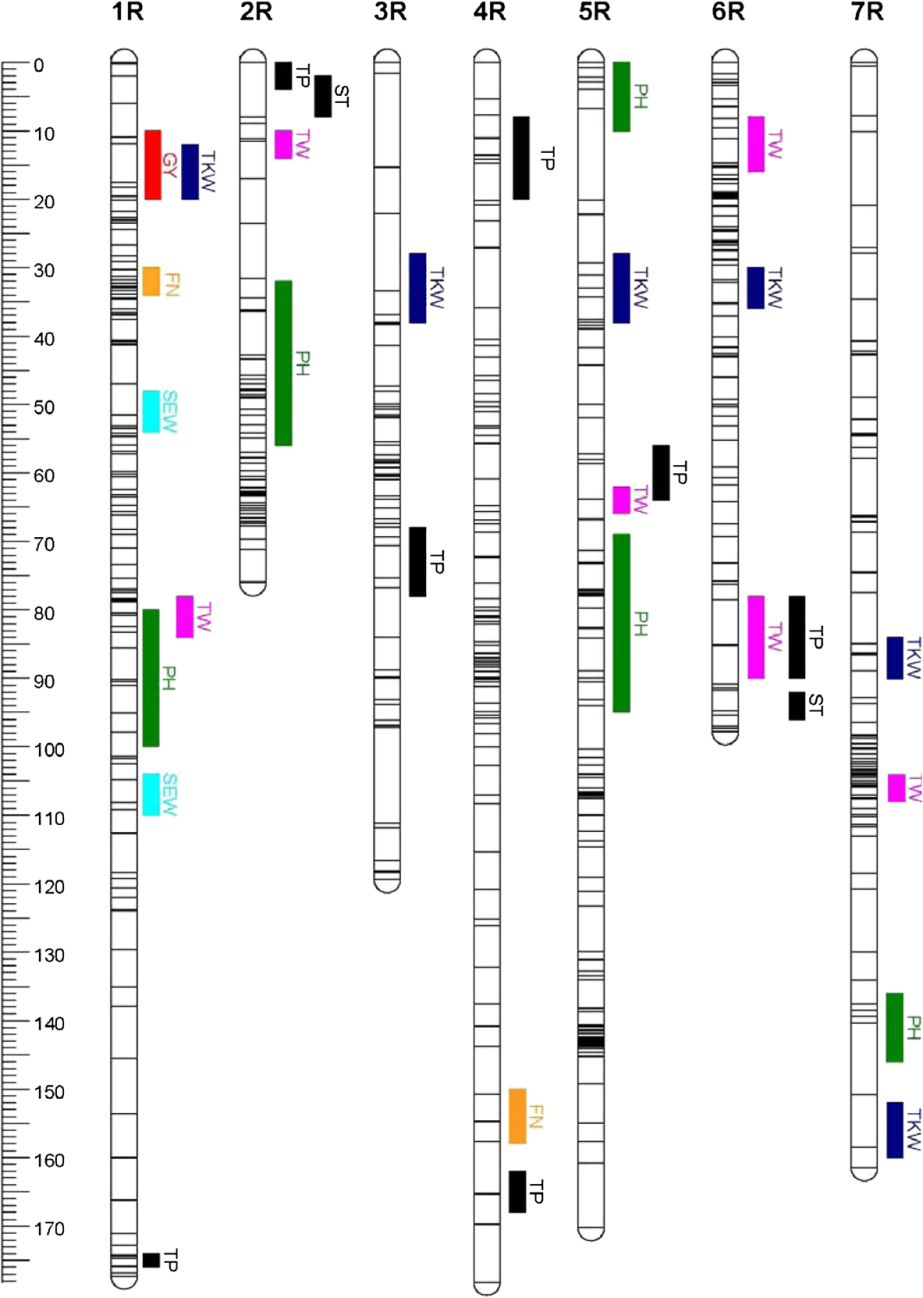
**Genetic linkage map of population A and detected quantitative trait loci (QTL).** Unique loci are represented on their positions by horizontal lines across the chromosomes showing 31 QTL distributed over 7 chromosomes (1R-7R). Support interval of QTLs are indicated by vertical bars; GY = Grain yield (red), PH = Plant height (dark green), TKW = 1000-kernel weight (blue), SEW = Single ear weight (turquoise), TW = Test weight (magenta), FN = Falling number (orange), TP = Total pentosan content (black), ST = Starch content (black).

**Figure 4:**
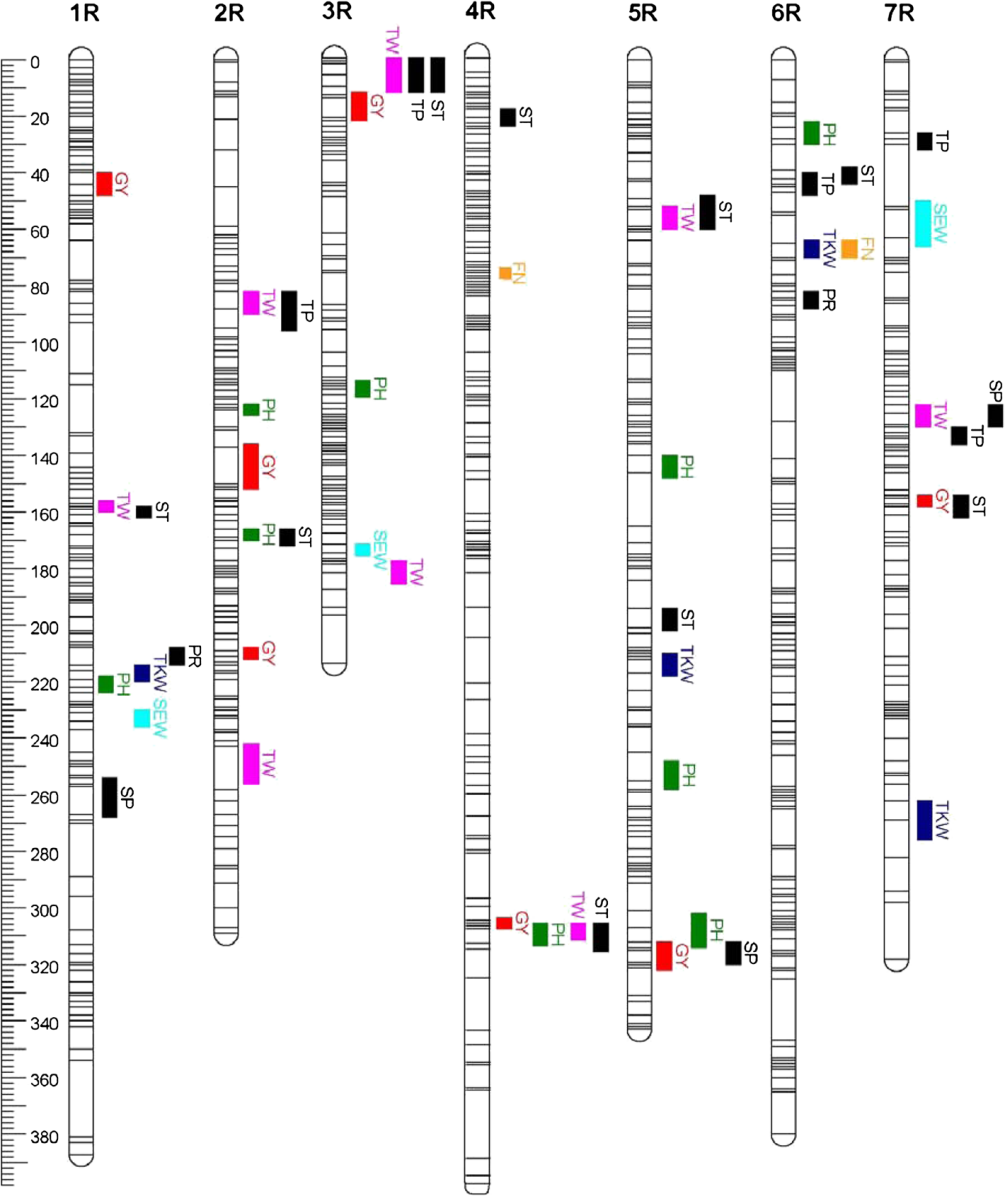
**Genetic linkage map of population B and detected quantitative trait loci (QTL).** Unique loci are represented on their positions by horizontal lines across the chromosomes showing 52 quantitative trait loci distributed over 7 chromosomes (1R-7R). Support interval of QTL are indicated by vertical bars; GY = Grain yield (red), PH = Plant height (dark green), TKW = 1000-kernel weight (blue), SEW = Single ear weight (turquoise), TW = Test weight (magenta), FN = Falling number (orange), TP = Total pentosan content (black), SP = Soluble pentosan content (black), PR = Protein content (black), ST = Starch content (black).

QTL analyses yielded one to nine QTL per trait, for soluble pentosan and protein contents no QTL were found in Pop-A (Table 3, for details see Additional files 4 and 5). Cross-validated phenotypic variances R^2^_CV_ were of similar order than the original R^2^_adj_ for all traits indicating a high quality mapping. QTL were distributed across all chromosomes (Figures 3 and 4). The only QTL for grain yield detected in Pop-A co-segregated with a QTL for TKW on chromosome 1R. For TKW, in each population two QTL with large effects were found on chromosomes 6 and 7 in Pop-A and on chromosomes 5 and 6 in Pop-B, respectively (Additional files 4 and 5). Some quality traits agreed in some of their QTL positions, especially in Pop-B.

**Table 3:** Summary of detected quantitative trait loci (QTL) after permutation test for ten traits

**Trait**	** No. QTL**	***R***^***2***^_***adj***_**(%)**		***R***^***2***^_***CV***_	***p***_**G**_**(%)**
		**Range**	**Total**		
	** Pop-A**				
Plant height	5	10-21	52.6	50.1	60.5
Yield-related traits:					
Grain yield	1	-	3.7	2.7	5.2
TKW	6	6-40	66.0	63.9	77.7
Single ear weight	2	8-9	16.2	14.5	49.1
Quality-related traits:					
Test weight	6	13-19	58.8	55.7	120.0
Falling number^a^	2	10-23	28.9	27.5	65.7
Total pentosan content	7	6-15	48.4	47.8	74.4
Starch content	2	14-24	46.9	46.1	61.0
	** Pop-B**				
Plant height	9	6-19	70.3	67.7	76.5
Yield-related traits:					
Grain yield	7	7-17	52.2	51.1	74.6
TKW	4	11-32	56.3	54.2	66.2
Single ear weight	3	7-10	26.1	23.2	50.1
Quality-related traits:					
Test weight	8	6-23	57.5	54.1	62.5
Falling number^a^	2	5-13	27.6	27.0	70.8
Total pentosan content	5	6-13	38.5	34.3	61.8
Soluble pentosan content	3	7-13	30.7	28.0	66.7
Protein content	2	8-10	17.2	15.7	23.2
Starch content	9	8-28	73.9	71.0	84.0

Number (no.) of QTL, proportion of explained phenotypic variance before (*R*^*2*^_*adj*_) and after cross validation (R^2^_CV_) and genotypic (*p*_G_) variance for each of 220 testcross progenies in two populations (Pop-A, Pop-B) estimated across ten (plant height and yield-related traits) and six (quality-related traits) environments.

^a^Analysis across 5 environments.

**Figure 5:**
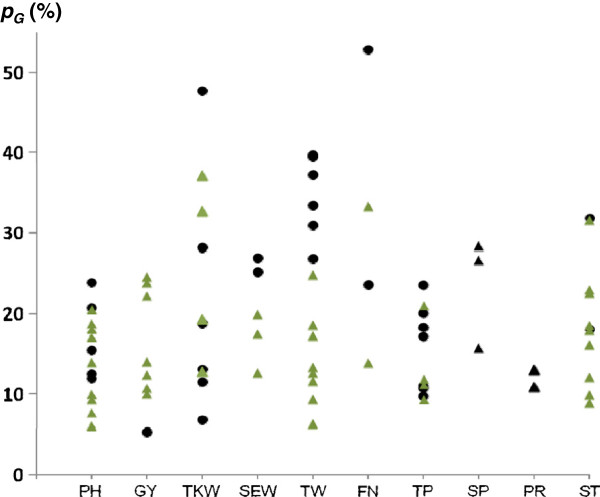
**Amount of genotypic variance (*p***_**G**_**) of each quantitative trait locus (QTL).** QTL were mapped for ten traits in population A (black dots) and B (green triangle). Values based on each of 220 testcross progenies per population estimated across ten (plant height and yield-related traits) and six (quality traits) environments. PH = Plant height, GY = Grain yield, TKW = 1000-kernel weight, SEW = Single ear weight, TW = Test weight, FN = Falling number, TP = Total pentosan content, SP = Soluble pentosan content, PR = Protein content, ST = Starch content.

The explained genotypic variance of the individual QTL (*p*_*G*_) ranged from 5 to 55% (Figure 5). The highest values of *p*_*G*_ were observed for TKW, test weight, and falling number. For these traits and starch content several QTL with large effects and a frequency of recovery of about 90% were identified in both populations (Additional files 4 and 5). Parents of the two crosses contributed equally to the detected QTL.

### Stability of QTL effects across environments

Most QTL revealed significant (P < 0.01) QTL × environment interaction variances as expected for quantitative traits (Additional files 4 and 5). The QTL × environment interaction variances, however, were in all cases smaller than QTL variance (data not shown). The identified QTL had a high stability across locations as illustrated for grain yield and TKW QTL (Table 4). They had high additive effects in at least nine out of ten environments.

**Table 4:** Additive effects within and across environments

**QTL**	**Chr.**	**Donor**	**Additive effect**										
			**2010**					**2011**					**Combined**
			**WOH**	**PET**	**WAL**	**BEK**	**HOH**	**WOH**	**PET**	**WAL**	**BEK**	**HOH**	
*Grain yield (Mg ha*^*-1*^*)*													
**Pop-A**													
1	1	Lo90	0.052	−0.034	0.206	−0.017	−0.500	0.101	0.112	0.005	0.061	0.063	0.054
**Pop-B**													
1	1	Lo117	0.085	0.028	0.379	0.052	0.107	0.100	0.003	0.013	0.029	0.019	0.087
2	2	Lo117	0.048	0.119	0.035	0.077	0.040	0.200	0.109	0.010	0.139	0.106	0.093
3	2	Lo117	0.100	0.066	−0.024	0.108	−0.093	0.106	0.100	0.009	0.027	0.247	0.064
4	3	Lo117	0.096	0.014	0.077	0.087	0.038	0.103	0.040	0.002	0.018	0.037	0.057
5	4	Lo117	1.094	0.161	0.070	0.155	0.109	0.035	0.128	0.008	0.065	0.051	0.089
6	5	Lo117	0.040	0.003	−0.040	0.061	0.011	0.158	0.071	−0.001	0.158	−0.013	0.052
7	7	Lo117	1.014	0.140	0.001	0.040	0.025	0.056	0.136	0.013	0.084	0.049	0.083
*1000 kernel weight (g)*													
**Pop-A**													
1	1	Lo90	0.347	0.035	0.823	0.352	0.208	0.299	0.280	0.139	0.184	0.265	0.291
2	3	Lo115	0.358	0.299	0.308	0.152	−0.169	0.241	0.374	0.546	0.407	0.155	0.269
3	5	Lo90	0.349	0.353	0.270	0.344	0.286	0.387	0.256	0.461	0.462	0.226	0.338
4	6	Lo90	0.354	0.536	0.518	0.461	−0.010	0.365	0.682	0.494	0.475	0.457	0.425
5	7	Lo115	0.594	1.152	0.747	0.527	0.135	0.670	0.759	0.701	0.717	0.623	0.659
6	7	Lo115	0.246	0.360	−0.028	0.047	−0.109	0.274	0.348	0.472	0.185	0.268	0.208
**Pop-B**													
2	1	Lo115	0.379	0.350	0.193	0.324	0.026	0.550	0.414	0.419	0.625	0.469	0.375
3	5	Lo117	0.697	0.515	0.161	0.748	0.050	0.781	0.460	0.422	0.910	0.546	0.529
4	6	Lo117	0.406	0.788	0.647	0.493	−0.124	0.655	0.624	0.711	0.628	0.749	0.557
5	7	Lo115	0.338	0.308	0.436	0.591	0.426	0.434	0.394	0.384	0.566	0.334	0.421

Effects of all detected quantitative trait loci (QTL) for grain yield (Mg ha^-1^) and TKW (g) for each of 220 testcross progenies in two populations (A, B) at ten individual environments and combined across environments.

## Discussion

We used two large segregating populations to unravel the genetic architecture underlying ten important agronomic and quality traits in rye. The results of the QTL mapping study opens for the first time comprehensive insights into the potential of marker-assisted selection (MAS) in rye.

### Field testing resulted in high-quality phenotypic data

The precise estimation of phenotypic values is an important prerequisite for detecting QTL with a high power. We used F_3:4_ lines and consequently, only 1.5 times the additive genetic variance was exploited and not 2 times the additive genetic variance as by using for instance recombinant inbred line populations. Due to the high inbreeding depression in the outcrossing species rye, testcross progenies of two elite line populations were used in this study. This allows exploiting only half of the total additive genetic variation, but takes into account that line *per se* performance is only of limited predictive value for hybrid performance in yield-related traits in rye [28]. Dominance is a serious obstacle when testcross performance is used in mapping biparental populations. When the tester contributes a strong dominant allele, the effect of the allele contributed by the other parent (an inbred line) is undetectable. The limitations on the use of strong elite testers were discussed by [29,30]. However, despite these limitations we observed significant genetic variation (P < 0.01) for all measured traits. The significance of genotype-by-environment interaction for all traits illustrate that multi-environmental phenotyping is indispensible. Entry-mean heritabilites reached 0.9 for plant height, TKW, and test weight in both populations and even grain yield had a heritability of 0.7. Lower heritabilities of some traits were mostly associated with a small, although significant amount of genotypic variation. Consequently, a high power to map QTL should be possible.

The parents in the Pop-A did not differ much in their testcross performance, which underscores a typical situation in elite rye breeding, which relies on crosses of the “best × best” type. In contrast, in Pop-B a superior (Lo115) and a lower performing (Lo 117) parent were crossed. Despite this, genotypic variance in Pop-A was similar (Figure 1) pointing towards a high importance of transgressive segregation. Obviously, both parents contributed different positive alleles at several loci for each of the traits. This is in accordance with quantitative-genetic theory assuming a large number of segregating loci with mainly additive effects for complex traits [31]. The contrast of the mean testcross performance of the parents and the average testcross performance of their segregating progenies is a test for net epistasis across the genome [32]. Consequently, the lack of differences between the mean performance of the parents and the progenies suggests that epistasis was not important in these populations. This can be explained by prevalence of additive gene action but might also be due to the fact that negative and positive effects at individual loci may cancel each other out.

### Plant height in elite rye populations is not controlled by major QTL

Plant height is regulated to a large extent by dwarfing genes in elite wheat and barley populations [33,34], because they have positive effects on elite wheat grain yield [35]. The genetic architecture of plant height in rye detected in this study, however, is in sharp contrast to the genetic architecture of plant height in elite wheat and barley. Five to nine QTL located on all seven chromosomes were identified for plant height in both elite rye populations. None of these QTL had a large genotypic effect and the recovery rates in permutation tests were low although the trait displayed a maximum heritability (0.9) and high proportions of explained genotypic variances (61 and 77% in Pop-A and Pop-B, respectively). This clearly illustrates that plant height in rye is a typical quantitative trait with a lot of segregating loci. Accordingly, [36] detected 11 genomic regions significantly contributing to plant height among testcrosses with two rye introgression line libraries. The main cause for the difference between wheat and barley on one hand, and rye on the other is that in rye the stem is used as reservoir for water and carbohydrates, especially when abiotic stress occurs. Very often grain yield is associated with tall plant stature as demonstrated by the significant correlation between plant height and grain yield in Pop-B (r = 0.30, P < 0.01). Therefore, no dwarfing gene has been successfully used in commercial rye cultivars until now although such a gene has been described and mapped [8]. Interestingly, we observed in Pop-B a QTL for plant height in the telomeric region of chromosome 5RL (QTL #8) where the *Ddw1* dwarfing gene from a Russian source was located (Figure 4, [8]). This QTL had in our population, however, a much smaller effect (R^2^ = 18.9) than expected from a major gene although the recovery rate was 96%, perhaps reflecting a multi-allelic series at this locus. In conclusion, quantitative inheritance of plant height in rye is an example for a highly crop-specific trait.

### Genetic architecture of yield and yield-related traits is generally complex, but some major QTL occur

Segregation at major effect QTL underlying grain yield in rye is not expected as any large-effect QTL would have been long fixed in the course of breeding. In agreement with this expectation, we observed for Pop-A where two high-yielding elite parents were crossed only one QTL explaining 5% of the genotypic variation (*p*_*G*_). In Pop-B deducted from a high- and a lower-yielding parent, seven QTL for grain yield were detected with *p*_*G*_ varying from 10 to 24% and low recovery rates. High effects in most of the tested individual environments reveal their environmental stability (Table 4). Accordingly, cross-validated variance explained by all QTL amounted to R^2^_CV_ = 51%. Here, linkage blocks due to limited resolution of QTL mapping in biparental populations may have lead to detection of clusters of linked QTL, thus underestimating the number of QTL involved in complex traits and overestimating their effects as shown by [37] in a comparison of a conventional F_3_ and an intermated F_3_ population.

Population size used was about 220 lines per population. This is similar to the US-nested association mapping (NAM) population [38] in maize, but an even greater size might be valuable for quantitative traits. For grain yield, [26] detected two QTL by analyzing 244 testcross progenies of maize, but up to seven QTL when regarding 976 progenies. We can, therefore, expect that the number of QTL estimated here might represent the lower limit of QTL segregating.

In conclusion, grain yield follows an infinitesimal model as already proposed by [39] and MAS for individual QTL seems not to be a realistic option. Yield components might identify better candidates. Indeed in Pop-A, where grain yield had only a minimum cross-validated R^2^ of 3% only, TKW resulted in 64%. In Pop-B this value was similar high for both traits (>50%). Interestingly, in both populations individual QTL for TKW with large effects were found: QTL on chromosomes 6 and 7 in Pop-A as well as on chromosomes 5 and 6 in Pop-B.

The most prominent QTL for TKW on chromosome 7R had a remarkably high *p*_*G*_ value of 48% and a recovery rate of 87% (Additional file 4). Interestingly, a major QTL on this chromosome was already reported in a Petkus population associated with the marker SCM40 [40]. This marker was located in the centromeric region of the chromosome [41] and might correspond to our QTL #5 for TKW in Pop-A. A second locus for this trait [40] was associated with SSR marker WMS5 located in the telomeric region of chromosome 5RL (renamed as GWM5, [4]) and might correspond to QTL #2 in Pop-B. The two other loci with high *p*_*G*_ values and recovery rates >90% were located in a similar region on chromosome 6 in both populations but contributed by different parents. These results illustrate that some QTL for TKW (#4 and #5 in Pop-A and #3 in Pop-B) have such high effects that they might be caused by single genes. Further fine mapping of multiple alleles per QTL in this region will be needed to test this hypothesis.

A similar localization of QTL was observed for some yield-related traits. Two QTL for grain yield on chromosome 1R (Pop-A) and 4R (Pop-B) had similar positions like each of one QTL for TKW and test weight reflecting the significant correlations between the traits. The corresponding QTL were contributed by the same parent in both instances. Thus, an indirect improvement of grain yield by MAS of individual QTL for yield components that have a higher recovery rate might be feasible.

For yield and yield-related traits we tested our mapping populations at ten environments including locations from North, East and South Germany as well as Poland. Compared to other QTL studies, this is a high number and broad range of environments and we report here only QTL with significant effects across all environments. This surely restricts the number of QTL, but enables us to detect only environmentally stable QTL that should be valuable also in environments not tested here. QTL for TKW and grain yield detected in the combined analysis had similar effects in most of the individual environments.

In summary, QTL effects for grain yield were mainly small as expected from theory, three QTL with high effects, however, were detected for TKW. They were highly stable across environments and had a high recovery rate in the cross validation and, thus, should be investigated further.

### Quality traits are regulated by a complex genetic network

Two to nine QTL were detected for each of the quality traits. Their *p*_*G*_ values were mostly >60%, starch content amounted even to 84% in Pop-B, although testing intensity was lower than for the yield-related traits. It should be noted that until now selection in practical breeding programs where the elite parents were derived from was solely based on falling number as a measure for pre-harvest sprouting. Despite its high heritability reported in earlier studies [42] it is a complex inherited trait [10,11]. In this study, we found each of two QTL for falling number in both populations and the QTL on chromosome 4 RL might correspond to a QTL already described [11].

Generally, quality traits are regulated by a complex genetic network resulting in phenotypic and pleiotropic interactions among the traits as shown for the maize nested association mapping (NAM) population [38]. Also in hybrid rye, significant correlations were detected among traits ranging from −0.2 to +0.6 (Table 1). In particular, QTL for starch content overlapped with QTL for grain yield on chromosomes 3R, 4R and 7R in Pop-B. Accordingly, both traits showed a significant (P < 0.01) phenotypic correlation (r = 0.32). On chromosomes 1R, 3R and 5R a QTL for test weight had a similar position than a QTL for starch content indicating that plumber kernels might have higher starch content. Significant (P < 0.01) correlations were indeed found between starch content and test weight. The negative correlation between starch and protein content in both populations (r = −0.6 and r = −0.7) is well known from other studies in cereals (Jansen, pers. commun.), but could not be explained by co-localization of QTL in our populations. A negative correlation between starch and TKW was also observed in durum wheat [43]. The authors state that end-use of the kernel is clearly influencing the physio-chemical kernel characteristics implying properties such as milling quality, kernel hardness, and kernel protein content. In rye, a high selection pressure is on large, plump kernels, because rye as a crop tends to low kernel size limiting flour yield.

In conclusion, each of one QTL with large effects (*p*_*G*_ > 20%) and high recovery rates (>90%) was found for test weight (QTL #2), falling number (#2) and starch content (#2) in Pop-A and starch content (#1) in Pop-B that should be considered for MAS.

## Conclusions

We provide in this study the first comprehensive QTL analysis in rye based on a high-density genetic map including about 900 markers per population. We detected one to nine QTL per trait. Because all traits were inherited quantitatively with a substantial amount of genotype-by-environment interaction, this number is most likely to be underestimated. Our results suggest that in rye the number of QTL segregating for quantitative traits is large even in biallelic populations and for traits with high heritability as previously shown in maize (e.g. [26,44]). These results explain the inefficiency of MAS in the improvement of quantitative traits controlled by many loci with small individual effects [45]. Meanwhile it is obvious, that only large-effect QTL, like those found here for TKW, test weight, falling number and starch content, could be candidates for successful MAS in practical breeding. For all other traits with a complex genetic architecture, genomic selection using markers located on the whole genome without selecting the most prominent ones might be more efficient [46].

## Competing interests

The authors declare that they have no competing interests.

## Authors’ contributions

TM and PW conceived the design of this study and coordinated the experiments, PW was responsible for material development and BS assisted in phenotyping. MH was the executive person in this study, including phenotyping, mapping procedure and statistical analyses, TM made the concept and wrote the manuscript. VK, EB, and GH were involved in marker genotyping. JCR applied his statistical knowledge, helped in designing the models and assisted in writing. All authors have read and approved the final manuscript.

## Supplementary Material

Additional file 1 **Estimates of variance components (genotypic, *σ***_***G***_^**2**^**; pooled error, *σ***_***e***_^**2**^**), and repeatabilities (Rep.) for 10 traits in 2010 and 2011 for Pop-A and Pop-B evaluated at five and three locations, respectively.** Click here for file

Additional file 2 Genetic linkage map of Pop-A for seven rye chromosomes with the distance in cM. Click here for file

Additional file 3 Genetic linkage maps of Pop-B for seven rye chromosomes with the distance in cM. Click here for file

Additional file 4 Quantitative trait loci of four agronomic and four quality traits in Pop-A. Click here for file

Additional file 5 Quantitative trait loci of four agronomic and six quality traits in Pop-B. Click here for file
