# Supplementary material for: Genetic architecture of complex agronomic traits examined in two testcross populations of rye (Secale cereale L.)
Source: BMC Genomics. 2012 Dec 17;13:706. doi: 10.1186/1471-2164-13-706 (PMC3566906; doi:10.1186/1471-2164-13-706)
Supplement: Additional file 1 — Estimates of variance components (genotypic, σG2; pooled error, σe2), and repeatabilities (Rep.) for 10 traits in 2010 and 2011 for Pop-A and Pop-B evaluated at five and three locations, respectively. [file 1471-2164-13-706-S1.pdf]

Additional file 1 - Estimates of variance components (genotypic, σ_G^2; pooled error, σ_e^2), and repeatabilities (Rep.) for 10 traits in 2010 and 2011 for Pop-A and Pop-B evaluated at five and three locations, respectively.

**Year**	**Pop-A**			**Pop-B**		
Environment	σ_G^2	σ_e^2	Rep.	σ_G^2	σ_e^2	Rep.
Grain yield (Mg ha^{-1})						
**2010**						
WOH	0.129**	0.079	0.58	0.184**	0.057	0.73
PET	0.163**	0.116	0.54	0.131**	0.083	0.57
WAL	0.130**	0.201	0.36	0.219**	0.065	0.74
BEK	0.091**	0.055	0.59	0.127**	0.050	0.68
HOH	0.200**	0.300	0.37	0.163**	0.417	0.25
**2011**						
WOH	0.090**	0.094	0.45	0.191**	0.131	0.56
PET	0.114**	0.080	0.56	0.122**	0.108	0.49
WAL	0.099**	0.143	0.47	0.158**	0.082	0.64
BEK	0.090**	0.121	0.39	0.069**	0.119	0.33
HOH	0.069**	0.187	0.24	0.107**	0.114	0.44
Plant height (cm)						
**2010**						
WOH	20.6**	8.0	0.69	28.9**	6.5	0.79
PET	19.7**	15.0	0.53	24.0**	14.0	0.60
WAL	9.7**	17.3	0.34	12.5**	8.1	0.57
BEK	14.2**	11.1	0.54	22.3**	6.7	0.74
HOH	17.1**	13.1	0.53	27.4**	14.3	0.62
**2011**						
WOH	23.3**	18.1	0.53	30.3**	7.7	0.78
PET	17.4**	16.9	0.47	14.5**	18.4	0.41
WAL	9.9**	14.3	0.37	26.7**	11.1	0.67
HAG	16.3**	14.1	0.50	21.8**	9.6	0.66
HOH	20.0**	16.1	0.52	29.8**	12.5	0.67
1000-kernel weight (g)						
**2010**						
WOH	1.5**	1.9	0.43	1.5**	0.6	0.70
PET	2.0**	0.6	0.73	1.6**	1.0	0.59
WAL	2.5**	3.2	0.40	2.8**	1.4	0.63
BEK	1.6**	0.9	0.62	1.8**	2.0	0.45
HOH	2.1**	1.1	0.61	2.1**	1.5	0.55
**2011**						
WOH	1.6**	1.1	0.55	2.6**	0.6	0.80
PET	1.6**	0.6	0.72	1.4**	0.8	0.62
WAL	3.0**	4.7	0.36	2.3**	4.4	0.31
HAG	1.8**	0.8	0.67	2.5**	0.7	0.77
HOH	1.1**	1.7	0.37	1.7**	0.7	0.67

Additional file 1 (continued)

**Year** Environment	**Pop-A** σ_G^2	σ_e^2	Rep.	**Pop-B** σ_G^2	σ_e^2	Rep.
Single ear weight (g)						
**2010**						
WOH	0.05**	0.02	0.42	0.01**	0.01	0.33
PET	0.01**	0.02	0.25	0.01**	0.02	0.27
WAL	0.02**	0.04	0.35	0.02**	0.04	0.26
**2011**						
WOH	0.01**	0.02	0.33	0.01**	0.02	0.34
PET	0.01**	0.03	0.21	0.004*	0.03	0.13
WAL	0.02**	0.03	0.35	0.01**	0.04	0.19
Test weight (g)						
**2010**						
WOH	0.9**	0.3	0.73	2.0**	0.3	0.84
PET	0.7**	0.2	0.72	1.1**	0.1	0.87
WAL	1.6**	1.0	0.59	2.2**	1.0	0.65
BEK	1.1**	0.2	0.83	1.5**	1.0	0.59
HOH	1.0**	0.5	0.65	0.8**	0.9	0.43
**2011**						
WOH	0.6**	0.5	0.53	1.0**	0.3	0.77
PET	0.5**	0.2	0.68	0.9**	0.3	0.72
WAL	0.7**	0.5	0.51	0.9**	0.5	0.62
BEK	0.6**	0.4	0.56	1.2**	0.3	0.80
HOH	1.6**	2.7	0.34	5.4**	1.6	0.75
Falling number (sec.)						
**2010**						
WOH	169.8**	300.5	0.35	143.4**	309.8	0.29
PET	179.1**	103.7	0.63	101.2**	67.6	0.57
HOH	606.7**	1383.1	0.27	379.9**	1187.7	0.21
