# Supplementary material for: Genetic architecture of complex agronomic traits examined in two testcross populations of rye (Secale cereale L.)
Source: BMC Genomics. 2012 Dec 17;13:706. doi: 10.1186/1471-2164-13-706 (PMC3566906; doi:10.1186/1471-2164-13-706)
Supplement: Additional file 2 — Genetic linkage map of Pop-A for seven rye chromosomes with the distance in cM. [file 1471-2164-13-706-S2.pdf]

Lo115xLo90 (Pop A) SSR, SNPs = 972 cM										
**nloc=164**			**nloc=91**			**nloc=71**			**nloc=119**	
**1R**	**cM**		**2R**	**cM**		**3R**	**cM**		**4R**	**cM**
c9696_667	0		c5115_94	0		c3970_1532	0		c12183_329	0
c28206_258	0,172		c14144_213	0		c45674_298	1,575		c12183_764	0
c3903_124	2,025		c11531_360	7,968		c13282_921	15,28		c5198_703	5,428
c4422_611	6,001		scm254xxxx	8,916		rems2085xx	22,108		rms1007xxx	7,703
c935_1675	10,884		c10409_1204	11,149		c49394_99	33,351		c7637_1495	10,962
c24346_153	11,884		c10409_352	11,149		c615_815	36,906		c7637_405	11,149
c3264_1316	17,604		c639_616	11,461		c11510_942	37,997		c9131_1942	13,577
c3264_1580	18,276		c11740_785	11,469		c9523_740	38,164		c30867_469	13,577
c5796_784	19,47		c2124_1364	11,481		c4179_600	38,333		c12942_961	13,577
c2960_331	19,552		c2124_1624	11,481		c16975_2735	41,35		c12942_952	13,577
c2787_590	19,562		c6930_1317	17,015		c24484_303	47,256		rems2879xx	14,146
c10977_872	20,103		rems2201xx	23,451		c5758_1660	47,996		c9131_1242	14,746
c16366_1582	21,807		rems2647xx	23,451		c552_1356	49,894		c13263_1054	20,205
c17804_212	21,814		c29063_266	31,627		c552_682	49,904		c12140_1217	20,8
c3162_1132	22,741		c3286_1367	34,363		c10351_1174	50,296		c8433_714	23,22
c25205_175	22,959		c59736_285	36,169		c17409_1226	50,658		c16256_705	27,018
c31398_988	23,171		c6950_376	36,384		c17409_976	50,658		c15845_566	27,226
c20066_368	23,433		c11525_1154	42,726		c2766_576	50,678		c1120_1377	35,937
rems2060xx	24,374		c56348_84	43,272		c5636_1473	51,494		c5049_539	40,502
rems2635xx	26,724		c9088_859	43,272		c18275_1464	51,826		c35736_212	41,449
scm271xxxx	28,252		c12069_802	45,679		c18275_1404	51,826		c8094_483	43,038
rems2155xx	29,089		c24453_251	46,335		c8091_580	51,992		c7465_517	45,819
rems2096xx	30,273		c9965_691	47,034		c7806_845	55,487		c3161_343	46,529
c6468_788	31,306		c1886_832	47,702		c10464_285	55,854		c30143_931	48,384
c6047_402	31,319		c1886_676	47,766		c20145_268	57,348		c13517_416	49,62
c6344_1248	31,766		c2510_372	47,88		c21344_711	58,015		c20655_491	50,298
c7576_398	31,783		c3313_470	47,88		rems2507xx	58,347		c1917_408	51,117
c9508_882	31,783		c14328_932	48,534		c6136_622	58,513		c15567_794	53,138
c1269_382	32,339		c29486_793	48,761		scm087xxxx	58,513		c577_913	53,428
c8793_450	32,344		c14328_998	49,031		c14759_1453	59,18		scm277xxxx	54,482
c5768_539	32,399		c14328_1282	49,031		c14759_1474	59,18		rems2329xx	55,66
c36606_493	32,759		c11957_204	50,662		c14759_1583	59,18		rems3028xx	60,924
c14487_256	32,78		c26275_224	51,574		c18316_225	60,189		c13294_167	64,84
c6684_693	32,78		c18426_2659	52,924		c6676_1452	60,354		c13294_893	64,84
c2018_566	32,78		c31322_354	52,924		c8896_2147	60,521		c19408_985	65,663
c18870_1400	32,808		c16939_805	54,148		rems2509xx	61,02		c9269_1176	66,86
c5803_1421	32,833		rems2438xx	54,939		c20565_792	63,403		c9941_1700	67,398
c5738_1677	33,007		c19935_372	57,021		c10803_1238	63,783		c6207_678	68,667
c7067_1946	33,252		c2160_541	57,572		c517_2111	65,169		c26374_277	68,683
c1062_470	33,277		c7971_248	57,749		c7601_1377	65,174		c11360_1327	72,11
c14934_542	33,776		c68207_288	57,751		c24995_160	65,178		c11360_1222	72,114
c6468_188	33,776		c7861_333	58,52		c11854_372	66,84		c2866_2065	72,314

c3177_1067	33,776		c18252_852	59,677		c11854_1006	67,259		rems3037xx	76,143
c3783_1642	33,776		c35842_115	59,677		c10748_413	67,864		c16119_273	78,301
c4989_261	33,776		c13726_464	59,677		c5362_414	69,378		c12096_283	78,325
c2909_795	33,776		c1513_821	59,677		c18177_127	70,599		c16119_315	78,331
c3783_909	33,776		c675_516	60,514		c1775_549	75,314		c1501_369	78,346
c18055_385	33,776		c8969_1471	60,514		c14032_1049	76,714		c3671_752	79,551
c3177_1506	33,776		c10643_674	61,013		c2631_1338	76,714		c3671_815	79,574
c13019_599	34,379		c36956_113	61,013		c6479_522	84,017		c7571_1271	80,112
c3579_323	34,379		c10572_727	62,02		c11290_2174	88,841		c4857_428	80,915
c7081_806	34,381		c6921_1723	62,02		c11290_1188	88,841		c6974_271	80,915
c13019_733	34,381		c14244_1303	62,02		c11290_1125	88,841		c3616_430	81,137
c13019_468	34,547		c1024_2021	62,02		c23752_566	89,827		c7800_322	81,567
c17932_604	34,608		c2899_1895	62,02		c4505_1034	90,042		c10371_1258	82,003
c25950_857	34,609		c3924_305	62,112		c7934_638	93,107		c6492_4206	84,66
c6945_2971	36,11		c2899_1055	62,185		c2842_625	93,814		c6492_6255	84,66
c8582_1036	36,465		c5982_1160	62,185		c6750_615	96,084		c11034_537	85,159
c7681_1946	36,465		c9831_2027	62,185		c4890_1726	96,89		c8025_3692	85,159
c3814_689	36,465		rems2232xx	62,684		c11025_395	96,89		c8990_4309	86,337
c10787_852	36,797		c1815_1327	62,707		c5691_953	97,222		c1719_1528	86,337
c22596_430	36,797		c1006_61	62,753		c5691_991	97,222		c1951_496	86,908
c60143_109	36,962		c8639_906	62,982		c27126_442	111,117		c1951_342	86,908
c6927_2385	37,63		c21669_1485	63,179		c7580_2193	111,845		c22580_519	87,08
c6927_2467	37,63		c21293_150	63,181		c50309_203	116,608		c25303_532	87,442
c5101_750	40,551		c1976_1202	63,291		c9413_340	118,055		c17403_240	87,442
c5101_444	40,551		c1770_1476	63,381		c2159_3284	118,063		c36846_231	87,666
c4242_890	40,703		c1692_252	64,352		c15647_155	118,063		c8212_102	87,666
c16620_714	40,821		c17666_682	64,373		rems2676xx	118,229		c12528_1931	87,998
scm021xxxx	41,069		c4865_1411	64,763		rems2073xx	118,394		c12528_1773	87,998
c16620_633	41,268		c19217_1112	65,185		rems2419xx	119,401		c3653_557	87,998
rems2427xx	47,041		c4822_357	65,185					c3601_533	87,998
c313_2926	51,478		c988_631	65,197					c11780_1659	88,042
c15445_443	51,481		c3263_423	65,479					c15269_404	88,299
c22090_408	51,501		c16497_653	65,484					c281_231	89
c22090_379	51,511		c34570_124	66,018					c22581_275	89
c1473_416	51,575		c1726_3339	66,507					c9834_652	89
c16340_271	53,083		c1726_1698	66,532					c12528_1008	89,908
c7809_2710	53,365		c1726_2564	66,598					c3698_994	90,066
c14590_620	53,419		c1726_2949	66,684					c7578_256	90,15
c14590_548	53,545		c1726_1959	66,684					c8130_812	90,158
rems2052xx	54,173		c65958_149	67,066					c6179_422	90,17
c14590_533	54,616		c10852_528	67,441					c25797_246	90,17
scm107xxxx	54,672		c18638_527	67,441					c1635_2164	90,17
c27981_213	54,672		c8569_538	67,827					c1635_2247	90,17
c16292_1378	55,85								c21571_274	90,57
c5700_757	56,868								c8349_2007	91,131
c12391_1254	56,872								c7578_258	91,229
c10696_1522	57,235								c10902_1380	93,633
c25550_691	59,752								c44356_158	94,924
c4903_1097	60,201								c2015_320	95,363
c416_1082	60,202								c736_796	95,808

c20350_500	60,635								c7693_328	96,601
c4809_1079	62,356								c12348_1073	98,11
c11228_272	63,198								c12348_1109	98,117
c7187_1089	63,45								c9581_1492	100,067
rems3190xx	64,667								scm079xxxx	102,727
c19610_728	65,73								c5393_2497	106,977
c8121_363	66,248								c1280_2221	106,977
c7202_162	68,208								c21705_178	106,977
c17419_237	68,997								c11405_198	106,977
c5048_250	70,923								c11405_213	106,977
c4204_593	70,974								c35371_803	108,346
c1502_726	73,278								c14490_1935	115,25
c1502_762	73,281								c2584_138	120,824
c8415_568	75,405								c3716_638	125,076
c8711_3534	75,405								rems2092xx	126,11
c12564_272	75,405								rems2738xx	132,175
c8711_3345	75,405								c6294_613	137,533
c19446_665	75,405								c15550_479	140,75
c11689_884	76,755								c15550_551	140,752
c5631_1506	77,087								c4335_574	143,781
c5686_927	77,419								rems2751xx	150,652
c10013_747	78,259								c41262_167	154,692
c26296_1414	78,425								c27613_860	157,735
c10013_772	78,59								c51957_304	165,284
c9030_204	78,792								c6612_1773	169,696
c26456_212	80,434								c8500_1414	169,696
c26456_464	80,481								rems2474xx	178,159
c8331_818	80,825									
c20745_159	82,418									
c3899_1669	83,183									
c1915_716	83,209									
c16840_1042	85,556									
c47126_265	85,556									
c16840_937	85,556									
c12509_464	90,185									
c12466_167	90,185									
c12509_604	90,204									
c12219_1001	90,516									
c14912_1997	91,017									
c14355_142	95,125									
c10735_694	97,876									
c3306_824	101,364									
c10593_841	101,683									
aif023xxxx	102,429									
c2833_1992	104,805									
c2246_416	108,088									
rms1107xxx	109,216									
c5573_1607	112,581									
aif038xxxx	118,428									
c25355_634	119,305									

rems2936xx	120,628									
c59423_201	122,045									
c33914_168	123,77									
c6428_286	123,962									
c10693_484	129,649									
rems2349xx	134,951									
c16457_126	137,892									
c16243_243	145,539									
c6354_1857	153,7									
c6449_1315	159,967									
c8781_583	166,247									
c6788_1707	171,109									
c28537_189	172,681									
c16115_1191	172,681									
c5903_298	174,204									
c11755_1147	174,204									
c5903_1910	174,446									
rems2326xx	174,701									
rems2225xx	174,701									
rems2307xx	175,886									
c9312_1221	176,753									
c9442_315	177,266									

	**nloc=124**			**nloc=129**			**nloc=115**	
	**5R**	**cM**		**6R**	**cM**		**7R**	**cM**
	c12589_779	0		c22015_431	0		c5789_372	0
	c3184_255	0,791		c31525_284	0		c11358_1083	0,521
	c25235_327	2,217		c2603_1837	1,697		c11358_1524	0,521
	c16530_935	2,24		c19823_409	2,536		c27076_262	7,771
	scm312xxxx	2,901		rems2495xx	3,036		c12588_690	10,1
	c11185_807	3,998		c18226_355	3,388		c7097_989	20,949
	c17904_128	6,823		c11263_2679	3,388		c9690_2177	20,949
	rems2083xx	20,065		rems2714xx	5,387		c9690_1856	20,949
	c9574_539	22,161		rems2703xx	6,452		c9962_731	27,004
	c24722_87	22,338		rems2613xx	8,172		c52181_231	27,767
	c5950_1023	29,3		c11498_2875	9,472		c2997_405	34,57
	rems2681xx	31,127		c4032_258	9,472		c14005_1202	40,723
	c2749_97	33,024		rems3155xx	11,131		c14939_719	42,073
	c2749_382	33,026		c11610_397	14,623		c17150_624	42,462
	c14308_437	34,329		c4092_1108	14,623		rems2340xx	42,744
	c4920_511	37,565		c6206_538	14,623		rems2843xx	48,943
	c4715_668	37,923		c26342_585	14,623		c12117_433	52,109
	c10854_413	38,385		c10268_93	14,674		c13527_878	54,228
	c4131_171	38,933		rms1090xxx	15,121		c13527_983	54,432
	c3472_1379	41,74		c19213_365	15,14		c13527_1220	54,644
	c12322_100	41,742		c3389_1603	15,158		rems2064xx	56,186
	c5180_3505	44,183		c15400_619	15,202		c11439_670	57,798
	rems3159xx	49,976		c4696_477	15,224		c5867_963	57,798
	c4778_2979	51,956		c36177_195	15,258		c24532_271	66,217
	c3036_849	57,246		c1425_700	15,286		c44283_421	66,296
	rems2248xx	57,962		c6771_1180	15,286		c9381_1005	66,386
	c11398_856	58,629		c4696_783	15,286		c15853_2049	66,386
	c8532_2900	58,629		c13470_436	15,286		c9381_1368	66,386
	c8532_2564	58,629		c19546_396	15,286		c20504_207	67,053
	c5496_455	58,629		c1333_1702	16,401		c14836_628	67,053
	rems2735xx	63,786		c1333_1508	16,401		c72890_62	68,663
	c23368_336	66,707		rems2542xx	17,131		c6065_292	74,39
	c6848_434	66,901		c6045_1965	17,798		rems2810xx	74,564
	c28485_426	71,316		c38529_199	17,798		c20611_314	77,394
	c12041_802	71,316		c3434_596	17,798		c19209_624	84,75
	c8267_898	73		c6270_1256	17,798		c27240_355	84,927
	c1379_675	73,187		c2080_1516	18,947		c7974_424	84,932
	c2091_475	76,864		c10948_335	19,015		c6557_853	86,253
	c1216_944	76,864		c10044_1885	19,14		c17682_548	86,508
	c1216_969	77,07		c4246_882	19,14		c4070_61	86,547
	c2091_257	77,195		c8714_258	19,144		rms1012xxx	88,891
	c2091_563	77,195		c7660_203	19,147		c1356_718	92,836

	c2244_2149	77,527		c2525_2213	19,164		c17727_117	93,746
	c5400_710	77,699		c1809_1181	19,19		rms1112xxx	96,376
	rems2365xx	77,858		c1523_1720	19,312		c10639_2309	98,274
	c2753_394	77,858		c5923_467	19,455		c956_1016	98,44
	c3219_798	79,701		c4696_465	19,682		c3142_513	98,443
	c12298_474	82,497		c10920_907	19,691		c2740_1151	98,445
	c13583_135	82,669		c10755_1043	19,898		c6734_498	98,455
	c63005_263	84,112		c6769_1008	19,905		c10683_1319	98,802
	c8377_339	88,86		c11911_464	20,866		c8088_1563	99,488
	c8732_461	90,007		c11911_352	20,967		scm063xxxx	100,094
	c40568_609	90,572		c5555_602	21,081		rems2098xx	100,26
	c40709_507	90,572		c1150_2505	21,081		c8482_1228	100,986
	c40568_600	90,575		c11733_146	22,455		c21856_542	101,145
	c9841_906	93,086		c6499_1372	22,461		c13257_1399	101,728
	c5884_1796	93,993		rems3090xx	24,028		c20375_659	102,054
	c13103_772	100,406		c19715_745	24,377		c19747_703	102,06
	c525_1109	101,527		c64951_94	24,377		c12154_1299	102,062
	c525_1075	101,577		c8934_1285	24,564		c6659_844	102,067
	rems2580xx	102,554		c7503_376	24,741		c21147_794	102,067
	c14516_1801	103,96		c1206_1995	25,998		c12638_962	102,072
	c8328_914	104,487		c1206_2328	26,059		c3054_1411	102,075
	c8381_458	105,894		c1206_1479	26,146		c20263_479	102,088
	c11064_767	106,594		c1206_1606	26,222		c21147_915	102,1
	c11769_585	106,678		c1206_1094	26,308		c3493_424	102,427
	c60812_223	106,764		c1206_3309	26,388		c3493_361	102,435
	c11695_1230	106,867		c1206_1003	26,388		c1223_480	102,758
	c6611_360	106,933		c12165_925	26,655		c23888_331	102,767
	c11294_741	107,037		c13035_630	27,432		c16122_560	103,237
	c23413_289	107,047		c2106_654	27,455		c6021_1566	103,351
	c4251_1295	107,05		c3822_414	27,467		c21605_465	103,528
	c4251_1703	107,058		c20151_161	27,561		rms1188xxx	103,754
	c11064_813	107,063		rems2324xx	28,732		c16520_864	103,937
	c14156_669	107,09		c9722_1019	28,937		scm019xxxx	104,085
	c49797_322	107,108		c21715_435	29,581		c19663_1040	104,388
	c23362_274	107,22		c5931_480	31,799		c415_728	104,886
	c15097_354	107,467		c15182_528	31,799		c6174_205	105,204
	rms1083xxx	110,041		c3026_1007	31,799		c9609_807	105,217
	c22500_246	112,279		c1961_918	31,829		c11707_1349	105,231
	rms1115xxx	113,652		c6514_1287	32,166		c23990_502	105,239
	c25640_138	114,563		c10911_727	32,176		c8227_1375	105,496
	c6401_553	119,119		c11417_584	35,124		c21063_177	105,512
	c7128_957	119,119		c18994_2228	35,336		c22825_582	105,651
	c235_261	121,093		c6290_2037	37,121		c8605_168	105,76
	rems2606xx	123,287		c17518_502	40,108		c6154_639	107,014
	scm141xxxx	129,935		c17518_285	40,108		c22645_1548	107,426
	c3599_901	131,116		c9647_1075	41,577		c22645_1122	107,426
	c3599_528	131,116		c1014_3335	41,696		c22645_1687	107,426
	c22565_981	132,782		rems2468xx	42,633		c4200_459	107,591
	c5254_2265	133,504		c40184_133	42,812		c43500_127	108,941
	c2288_628	134,015		c11417_363	43,012		c14129_884	108,941

	c1336_681	138,196		c10267_1044	45,981		c2981_266	109,776
	c2484_184	138,737		c2861_681	49,244		c13833_224	110,055
	c6032_341	138,737		c10865_428	49,969		c2128_559	110,06
	c9212_889	140,61		c10865_704	49,969		c22289_596	110,063
	c9212_306	140,61		c4063_300	50,31		c9768_3164	110,065
	c4508_729	140,775		c7023_764	51,69		c13305_997	111,349
	c4508_795	140,775		c7405_563	53,054		rems3015xx	111,623
	c4836_1139	140,941		c35975_273	55,16		c4215_328	112,954
	c9802_1146	141,349		c2256_811	59,125		c26527_269	118,411
	c9877_440	141,533		scm135xxxx	60,712		c5020_1423	118,413
	c10336_1496	141,765		c10581_200	61,774		c5020_1505	118,418
	c6552_839	141,778		c35547_326	64,231		c29065_160	120,684
	c14685_216	141,945		c12190_720	67,381		c23747_1185	129,951
	c1553_1221	142,383		c3467_222	69,341		c12562_2101	134,012
	c2988_1087	142,629		c2371_416	69,341		c12562_2590	134,012
	c11720_299	142,775		c2108_427	73,091		c12562_1772	134,012
	c12607_267	142,985		c5392_472	73,091		rms1018xxx	137,459
	c13504_383	143,221		c32149_446	75,784		c6372_400	138,367
	c1079_2000	143,438		c32149_406	75,788		c12187_1564	139,348
	c5416_3199	143,438		c12618_1087	75,788		c11925_1294	140,325
	c8878_2050	143,603		c1571_1132	76,326		rems2290xx	150,806
	c12696_279	143,768		rems2528xx	78,514		c10815_915	158,4
	c67276_87	143,934		c14562_499	78,514		c11139_144	161,489
	c13033_242	144,099		rems3192xx	78,535			
	c13033_590	144,099		rems2154xx	85,128			
	c5708_1115	144,646		rems2077xx	85,294			
	c5391_845	145,199		c2794_1223	90,852			
	c12595_1008	149,165		c14599_562	91,395			
	c5363_344	154,906		c13262_1887	91,395			
	c13042_585	157,679		c5320_1179	91,727			
	c3661_432	160,773		c20475_449	94,838			
	c14666_1717	170,224		c5780_590	95,355			
				c3864_1245	95,355			
				c8351_379	95,355			
				c16021_485	97,024			
				c24656_341	97,28			
				c10497_1433	97,78			
