# Supplementary material for: Genetic architecture of complex agronomic traits examined in two testcross populations of rye (Secale cereale L.)
Source: BMC Genomics. 2012 Dec 17;13:706. doi: 10.1186/1471-2164-13-706 (PMC3566906; doi:10.1186/1471-2164-13-706)
Supplement: Additional file 3 — Genetic linkage maps of Pop-B for seven rye chromosomes with the distance in cM. [file 1471-2164-13-706-S3.pdf]

**Lo115xLo117 (Pop B) DArT, SSR = 2345 cM**										
**nloc=137**			**nloc=124**			**nloc=120**			**loc=152**	
**1R**	**cM**		**2R**	**cM**		**3R**	**cM**		**4R**	**cM**
rPt-509417	0		rPt-507609	0		rPt-411037	0		rPt-507164	0
rPt-507103	3,162		rPt-508811	1,232		rPt-509549	0,364		rPt-398637	4,622
rPt-505878	4,775		rPt-507767	1,383		rPt-401430	0,98		rPt-390553	7,441
rPt-508726	5,241		rPt-508408	8,198		rPt-401028	1,551		rPt-506146	10,377
rPt-401572	6,904		rPt-505500	11,222		rPt-507113	1,871		rPt-401485	11,679
rPt-506579	8,257		rPt-390052	11,918		rms1254xxx	5,715		rPt-400726	13,244
rPt-402670	8,85		rPt-505391	12,327		rPt-402334	10,358		rPt-506330	14,433
rPt-508226	10,607		rPt-399568	13,243		wPt-345565	12,542		tPt-7135	15,745
rPt-506161	11,796		rPt-507495	20,811		rPt-400346	13,17		rPt-505440	16,625
rPt-400354	15,068		rPt-399876	32,33		tPt-8002	13,281		rPt-402514	18,065
rPt-400441	17,091		rPt-411511	44,822		wPt-344792	13,481		rPt-390550	20,756
rPt-401218	19,327		rPt-389683	59,26		rPt-7349	13,67		rPt-399682	21,156
rPt-390604	20,065		rPt-509238	61,705		tPt-5129	13,915		rPt-398656	23,333
rPt-507802	23,906		rPt-506496	62,642		wPt-344800	14,161		wPt-8814	24,492
rPt-509009	25,079		rPt-401244	64,533		tPt-1103	14,194		wPt-0562	25,448
rPt-389469	26,424		rPt-509676	66,851		rPt-505909	14,421		rPt-6659	27,152
rPt-410760	28,419		rPt-411008	68,932		rPt-400833	21,368		rPt-400701	30,012
rPt-398554	29,29		rPt-508312	72,96		rPt-400475	21,566		rPt-400310	32,241
rPt-507281	30,533		rPt-507168	74,609		rPt-509131	21,703		rPt-402250	33,327
rPt-509704	30,771		rPt-509105	74,681		rPt-505751	24,432		rPt-508638	35,244
rPt-400552	31,686		rPt-506443	75,213		rPt-507180	26,189		rPt-401308	38,416
rPt-507311	34,497		rPt-399610	78,436		rPt-398703	28,06		rPt-401070	38,421
rPt-507120	37,152		rPt-400973	79,201		rPt-508069	29,3		rPt-390409	39,54
rPt-400300	38,956		rPt-509246	82,08		rPt-505911	29,66		rPt-507540	39,607
rPt-399803	40,267		rPt-400938	88,248		rPt-400690	31,15		rPt-399841	41,145
rPt-508032	44,127		rPt-9914	94,972		rPt-509479	32,582		rPt-509365	42,512
rPt-390314	47,823		rPt-508543	95,013		rPt-401768	33,554		rPt-506231	47,084
rPt-399662	49,956		rPt-411470	95,076		rPt-508029	36,241		rPt-505672	48,281
rPt-506493	50,534		rPt-389972	97,642		rPt-401138	44,058		rPt-505828	48,664
rPt-507848	51,309		rPt-398690	98,709		rPt-401143	44,523		rPt-505181	50,323
rPt-400359	52,583		rPt-507737	98,965		rPt-399875	47,412		rPt-402360	51,553
rPt-400361	52,859		rPt-399644	99,341		rPt-400780	48,905		rPt-390135	53,497
rPt-506276	53,79		rPt-411208	100,879		rPt-505544	62,057		rPt-400857	55,083
rPt-507452	54,511		rPt-411501	102,671		rPt-505227	62,329		rPt-401943	56,099
rPt-506878	56,382		rPt-509381	102,697		rPt-399980	66,013		rPt-399899	57,312
rPt-402536	57,573		rPt-506972	104,824		rPt-400544	70,328		rPt-401890	59,065
rPt-505528	63,883		rPt-509001	109,149		rPt-508672	70,746		rPt-400824	59,753
rPt-389517	78,015		rPt-411506	110,259		rPt-401119	75,182		rPt-508305	61,184
rPt-505890	78,675		rPt-508204	110,879		rPt-507575	75,862		rPt-402104	65,437
rPt-402026	80,642		rPt-398639	113,073		tPt-3022	76,354		rPt-506088	66,573
rPt-411229	82,009		rPt-400888	114,359		rPt-506455	86,797		rPt-399902	68,54

rPt-402362	86,378		rPt-507031	114,932		rPt-509013	88,553		rPt-506948	71,943
rPt-399883	89,974		rPt-389716	117,242		rPt-401653	88,761		rPt-506089	73,015
rPt-399892	93,015		rPt-401233	119,075		rPt-9239	88,879		rPt-401670	73,47
rPt-507474	110,706		rPt-5439	120,155		rPt-508819	92,076		rPt-401280	74,31
rPt-508034	110,857		rPt-505566	121,808		rPt-507467	92,45		rPt-508800	75,244
rPt-508370	111,148		rPt-402474	122,673		rPt-507801	92,794		rPt-506376	75,702
rPt-508039	114,66		rPt-507837	123,119		rPt-4257	95,879		rPt-400382	76,227
rPt-509168	131,614		rPt-9132	123,528		rPt-402134	103,917		rPt-9258	76,975
rPt-505283	132,845		rPt-505263	124,172		rPt-505738	108,815		rPt-402215	77,216
rPt-505927	138,62		tPt-7975	124,338		rPt-506051	113,184		rPt-505373	77,239
rPt-398748	143,75		rPt-1348	129,507		rPt-390658	113,737		rPt-507289	78,322
rPt-411466	146,015		rPt-506760	130,879		rPt-508331	113,984		rPt-398763	79,09
rPt-399643	147,612		rPt-402660	136,958		rPt-509627	114,314		rPt-390787	79,198
rPt-508657	149,763		rPt-399303	150,366		rPt-399830	115,08		rPt-402101	79,314
rPt-508822	152,091		rPt-508897	150,795		rPt-401359	115,203		rPt-401183	79,323
rPt-508160	155,381		rPt-411226	150,905		rPt-399732	116,419		rPt-389501	79,403
tPt-5537	156,714		rPt-401797	152,36		rPt-400480	117,381		rPt-400484	80,235
rPt-505823	157,21		rPt-506524	152,398		rPt-399627	118,536		rPt-508260	80,812
scm004xxxx	158,18		rPt-507982	154,743		rPt-508194	122,082		rPt-402157	81,56
scm107xxxx	159,336		rPt-0722	156,462		rPt-506967	122,305		rPt-400520	81,954
rPt-509169	160,865		rPt-507750	158,047		rPt-400319	124,173		rPt-400996	82,583
rPt-505686	163,636		wPt-6877	161,127		rPt-508377	125,925		rPt-400835	83,59
rPt-509253	165,469		rPt-507927	162,51		rPt-411316	127,386		rPt-411080	90,662
rPt-507504	167,926		rPt-398641	166,016		rPt-411260	127,919		rPt-400064	91,97
rPt-399799	171,777		rPt-400282	168,657		rPt-401211	127,988		rPt-399387	93,02
rPt-509310	173,372		rms1042xxx	170,073		rPt-390014	128,743		rPt-400070	93,037
rPt-402616	174,806		rPt-402581	172,647		rPt-509361	129,78		rPt-507229	94,174
rPt-402546	175,169		rPt-507714	176,948		rPt-507729	129,904		rPt-507984	94,804
rPt-507342	175,541		rPt-505933	179,374		rPt-505215	130,75		rPt-506439	95,622
rPt-401076	175,569		rPt-402421	179,944		rPt-401940	132,333		rPt-399573	103,716
rPt-399930	176,415		rPt-399800	181,152		rPt-401113	134,051		rPt-401313	110,992
rPt-401335	177,039		rPt-507619	181,974		rPt-8081	135,552		rPt-508188	112,915
rPt-390587	180,31		rPt-508718	183,298		rPt-505832	136,63		rPt-509707	113,382
rPt-506666	183,387		rPt-411158	186,525		rPt-507717	138,141		rPt-508914	113,792
rPt-399592	184,662		rPt-508213	187,693		rPt-411113	139,37		rPt-402392	116,312
scm021xxxx	186,168		rPt-401533	188,356		rPt-509732	139,814		rPt-410824	118,797
rPt-508367	188,734		rPt-3473	188,821		wPt-3484	141,811		rPt-507894	120,282
rPt-401581	188,991		rPt-506904	188,939		rPt-398525	143,02		rPt-506357	121,318
rPt-505196	189,851		rPt-507665	192,979		rPt-506103	144,313		rPt-400124	122,701
rPt-390080	190,756		rPt-506408	195,378		rPt-401081	148,499		rPt-505900	123,312
rPt-508357	192,435		rPt-401707	196,553		rPt-398502	148,597		rPt-509554	128,962
rPt-398813	196,622		wPt-1470	197,118		rPt-401227	150,937		rPt-400750	133,544
rPt-401356	201,802		rPt-507787	198,647		rPt-505433	151,45		rPt-509435	135,828
rPt-509474	202,686		rPt-390224	203,366		rPt-506742	151,699		rPt-505620	136,307
rPt-507968	205,757		rPt-5429	205,307		rPt-505883	151,96		rPt-401029	140,018
rPt-411471	206,838		rPt-398762	209,115		rPt-399710	151,997		rPt-399468	141,463
rPt-508906	208,415		rPt-506176	210,933		rPt-506374	152,024		rPt-506645	145,753
rPt-3206	213,709		rPt-400410	212,542		rPt-505482	152,045		rPt-2478	145,781
rPt-401334	215,877		rPt-507323	213,579		rPt-509335	152,067		rPt-505288	149,374
rPt-400192	216,487		rPt-509383	214,491		rPt-401488	152,363		rPt-401169	160,931

rPt-507078	218,661		rPt-399280	215,995		rPt-505248	152,791		rPt-505674	161,083
rPt-508252	219,378		rPt-507867	216,811		rPt-398705	155,303		rPt-400820	163,876
rPt-506503	219,414		rPt-401315	217,319		rPt-410986	155,809		rPt-508292	164,486
rPt-400034	222,277		rPt-410800	219,052		rPt-399632	156,017		rPt-507812	166,971
rPt-400103	224,368		rPt-402364	224,614		rPt-505278	156,528		rPt-509552	168,179
rPt-508191	226,648		rPt-389503	226,075		rPt-401223	156,982		rPt-398573	171,127
rPt-508375	228,133		rPt-505627	228,971		rPt-410872	157,21		rPt-401010	171,577
rPt-509098	228,749		rPt-509630	230,227		rPt-509695	157,71		rPt-402563	171,76
rPt-509362	231,232		rPt-390410	231,988		rPt-400162	159,547		rPt-389394	173,06
rPt-402055	234,128		rPt-508235	232,72		rPt-506874	159,952		rPt-389569	174,441
rPt-508079	237,214		rPt-411512	233,115		rPt-509281	160,983		rPt-401509	176,415
rPt-401887	245,272		rPt-508490	237,227		rPt-400159	161,14		rPt-402560	176,532
wPt-343920	247,576		rPt-398735	238,151		rPt-390606	162,472		rPt-509255	176,548
rPt-505309	248,609		rPt-509610	240,912		rPt-389676	163,494		rPt-508199	182,059
rPt-401730	250,419		rPt-399333	242,651		rPt-402504	164,517		rPt-389930	193,727
rPt-507905	253,121		rPt-402236	243,004		rPt-507337	167,573		rPt-507671	204,546
rPt-400987	254,367		rPt-400854	257,716		rPt-400204	167,864		rPt-507094	220,562
rPt-410907	255,588		rPt-401657	257,817		rPt-399628	168,051		rPt-506976	226,523
rPt-401014	256,684		rPt-400975	261,904		rPt-506740	168,178		rPt-508404	238,56
rPt-402069	256,964		wPt-0271	266,908		rPt-402485	172,447		rPt-508577	239,008
rPt-411308	267,155		rPt-389862	271,203		rPt-4378	174,853		rPt-389872	242,517
rPt-410988	268,818		rPt-506245	275,402		rPt-7156	177,268		rPt-508623	243,171
rPt-507636	270,017		rPt-399784	279,007		rPt-506171	178,26		rPt-508467	247,067
rPt-508150	288,681		rPt-401202	284,98		rPt-410952	179,273		rPt-508910	249,466
rPt-399764	296,022		rPt-402355	285,887		scm294xxxx	181,57		rPt-389603	253,253
rPt-389336	308,12		rPt-400997	286,253		rPt-389353	188,172		rPt-7872	256,937
rPt-507059	312,604		rPt-399788	286,473		rPt-505214	193,729		rPt-389701	259,583
rPt-402080	313,024		rPt-401184	290,96		rPt-505591	197,028		rPt-508833	268,214
rPt-400374	315,893		rPt-505781	299,581		rPt-506858	213,739		rPt-507277	268,304
rPt-508520	318,956		rPt-506394	306,761					rPt-509321	274,77
rPt-505873	319,523		rPt-506621	306,927					rPt-506862	276,27
rPt-401180	320,02		rPt-505481	307,196					rPt-508998	279,741
rPt-400845	321,888		rPt-401952	308,878					rPt-506239	281,086
rPt-390364	326,11								rPt-506530	281,402
rPt-3268	330,326								tPt-4576	296,796
rPt-509600	331,345								rPt-507178	300,248
rPt-389334	332,521								rPt-506954	304,633
rPt-506577	335,124								rPt-508519	306,008
rPt-508703	338,161								rPt-508864	306,025
rPt-400754	339,786								rPt-506899	306,043
rPt-507090	342,196								rPt-507855	306,066
rPt-509262	350,233								rPt-507170	306,084
rPt-509102	353,975								rPt-506011	306,103
rPt-508808	380,607								rPt-509110	306,156
rPt-507765	382,522								rPt-509459	306,378
rPt-400928	386,712								rPt-508932	306,737
									rPt-508033	307,725
									scm352xxxx	313,048
									rPt-506669	314,952
									rPt-506436	325,226

									rPt-509077	344,286
									rPt-390721	349,279
									rPt-399906	355,233
									rPt-389466	356,168
									rPt-400301	364,342
									rPt-398700	364,442
									rPt-2245	364,853
									rPt-506940	364,873
									rPt-507653	389,322
									rPt-508456	395,022
									rPt-505548	398,306

	nloc=128			nloc=149			nloc=111	
	5R	cM		6R	cM		7R	cM
	rPt-506433	0		rPt-507326	0		rPt-401781	0
	rPt-508585	0,029		rPt-506274	0,024		rPt-402431	0,855
	rPt-390233	0,061		rPt-509663	7,133		rPt-401348	0,884
	rPt-399984	0,094		rPt-389362	15,026		rPt-506930	10,893
	scm312xxxx	8,216		rPt-506956	19,229		rPt-506974	11,667
	tPt-8896	9,394		rPt-505807	20,44		rPt-400851	13,696
	tPt-1893	9,524		rPt-507510	23,865		rPt-508478	17,15
	rPt-411270	15,271		rPt-507491	27,798		rPt-411363	18,136
	rPt-399953	19,35		rPt-509263	29,78		rPt-401972	26,063
	rPt-506113	20,517		rPt-508240	38,608		rPt-508316	27,521
	rPt-390522	22,503		rPt-401102	41,771		rPt-402501	28,215
	rPt-505480	22,543		rPt-411507	42,176		rPt-400783	28,226
	rPt-411267	24,192		rPt-401349	44,307		rPt-509329	28,299
	rPt-390260	25,656		rPt-505604	44,909		wPt-6821	30,242
	rPt-398767	26,761		rPt-401305	46,786		rPt-508646	51,701
	rPt-398506	27,305		rPt-401114	54,411		rPt-506137	52,323
	rPt-399681	28,44		tPt-6200	55,379		rPt-506208	52,682
	rPt-505228	32,704		rPt-507486	65,068		rms1187xxx	62,795
	rPt-399811	33,456		rPt-507211	65,076		rPt-508879	69,677
	rPt-508834	35,75		rPt-6603	65,108		rPt-411499	69,998
	rPt-507664	35,776		rPt-4601	65,135		rPt-402476	70,581
	rPt-507948	41,883		rPt-389829	69,718		rPt-507890	71,2
	rPt-505712	43,073		rPt-401736	70,521		rPt-507379	71,828
	rPt-505693	48,764		rPt-506066	76,162		rPt-507403	75,075
	rPt-508587	52,429		rPt-390498	78,588		rPt-508642	83,846
	rPt-506733	53,195		rPt-505867	80,262		rPt-401828	85,374
	rPt-7348	58,939		wPt-0058	81,998		rPt-411254	85,754
	rPt-508768	59,956		rPt-506395	83,947		rPt-508579	94,011
	rPt-508816	61,183		rPt-400398	84,581		rPt-505382	94,581
	rPt-389261	64,457		rPt-390263	85,372		rPt-402262	96,079
	tPt-8940	72,408		rPt-505956	87,104		rPt-410875	97,596
	rPt-410783	72,484		rPt-389437	89,659		rPt-508263	97,772
	rPt-402045	73,125		rPt-505347	91,004		rPt-509268	103,266
	rPt-410924	76,154		rPt-507635	91,034		rPt-401098	103,902
	rPt-508364	79,569		rPt-509473	91,043		rPt-402405	106,066
	rPt-508420	80,324		rPt-402654	91,291		rPt-506260	106,291
	rPt-400903	81,051		rPt-509477	91,555		rPt-508008	107,971
	rPt-401058	88,807		rPt-505912	97,768		rPt-508953	108,011
	rPt-508445	88,877		rPt-506668	100,126		rPt-507970	109,597
	rPt-410948	89,143		wPt-9790	100,326		rPt-399775	111,21
	rPt-509673	91,205		rms1250xxx	102,373		rPt-400878	112,166

	rPt-508041	91,497		rPt-410796	102,928		rPt-505462	114,664
	rPt-402367	92,77		rPt-390711	103,465		rPt-402059	116,785
	scm137xxxx	94,249		rPt-508055	104,751		rPt-506121	118,778
	rPt-507000	95,367		rPt-399922	105,16		rPt-410884	118,843
	rPt-401095	99,042		rPt-400777	106,405		rPt-410779	122,248
	rPt-402329	102,375		rPt-399561	107,23		rPt-399849	124,836
	rPt-505515	104,047		rPt-410892	108,122		rPt-401220	128,944
	rPt-507953	112,901		rPt-398480	108,813		rPt-505397	130,188
	rPt-507366	114,147		rPt-505711	109,54		rPt-505678	130,188
	rPt-411522	119,605		rPt-508389	127,662		rPt-390600	130,188
	rPt-401929	120,769		rPt-506193	140,943		rPt-506949	130,188
	rPt-411320	121,316		rPt-7238	148,495		rPt-402256	131,699
	rPt-411109	121,59		rPt-506184	149,172		rPt-390556	133,207
	rPt-390510	126,054		rPt-509216	150,461		rPt-508594	134,217
	rPt-398773	128,197		rPt-506554	156,598		rPt-508956	135,779
	rPt-508485	129,321		rPt-508341	158,762		rPt-505855	136,84
	rPt-399488	129,916		rPt-411144	160,684		rPt-410979	137,456
	rPt-506747	132,175		rPt-508835	162,505		scm019xxxx	137,507
	rPt-507137	132,907		rPt-399755	173,192		rPt-402657	138,405
	rPt-6165	133,045		rPt-509735	173,205		scm050xxxx	139,697
	rPt-508201	135,478		rPt-507032	173,434		rPt-410797	140,435
	rPt-400837	135,871		tPt-3774	174,681		rPt-399642	143,161
	rPt-411050	136,109		wPt-3915	176,575		rPt-389777	144,402
	wPt-344500	140,31		rPt-401505	186,881		rPt-400194	146,103
	rPt-389787	146,485		rPt-401477	188,119		rPt-400252	151,579
	rPt-402396	165,214		rPt-506052	189,169		rPt-506494	153,591
	rPt-507724	170,781		rPt-400788	189,38		rPt-505931	154,536
	rPt-6124	174,658		scm055xxxx	192,453		rPt-508118	155,459
	rPt-508209	176,036		rPt-401549	196,138		rPt-508571	157,491
	rPt-505650	176,706		rPt-389305	196,156		rPt-509598	157,737
	tPt-5290	178,619		rPt-401125	196,45		rPt-506183	157,954
	rPt-410942	179,519		rPt-390501	197,356		rPt-508630	161,325
	rPt-507355	183,534		rPt-411086	198,962		rPt-506764	166,728
	rPt-509532	193,809		rPt-400921	199,747		rPt-508837	168,92
	scm098xxxx	200,57		rPt-399635	202,938		rPt-402315	171,081
	rPt-508912	203,333		rPt-507896	205,46		rPt-401949	172,448
	rPt-505220	207,79		rPt-399834	207,358		rPt-390127	176,821
	rPt-507926	208,71		rPt-390171	209,22		wPt-346084	182,057
	rPt-507887	208,835		rPt-411128	211,747		rPt-508164	186,27
	rPt-6661	210,157		rPt-399540	212,228		rPt-508071	187,199
	rPt-506729	211,198		rPt-507463	214,399		rPt-505187	187,622
	rPt-506860	211,202		scm078xxxx	215,24		rPt-402418	187,666
	rPt-401688	211,701		scm295xxxx	215,393		rPt-390051	188,442
	rPt-399606	217,144		rPt-508919	217,713		rPt-401578	190,302
	rPt-509399	223,136		rPt-506263	223,758		rPt-508345	195,802
	rPt-400876	229,349		rPt-399825	223,775		rPt-505934	196,415
	rPt-400326	229,364		rPt-508321	223,786		rms1018xxx	201,203
	rPt-402532	229,813		rPt-399879	223,915		rPt-508904	211,127
	rPt-508427	234,608		rPt-507589	224,048		rPt-410913	213,979
	rPt-506172	236,434		rPt-508786	228,142		rPt-508402	217,846

	rPt-505770	245,435		rPt-390712	233,952		rPt-401423	221,244
	scm141xxxx	254,843		rPt-402383	237,743		rPt-505136	227,429
	rms1115xxx	257,819		rPt-399611	241,463		rPt-400278	227,974
	rms1083xxx	258,937		rPt-509058	241,539		rPt-505647	228,345
	rPt-0049	264,201		rPt-506500	245,894		rPt-399735	228,755
	wPt-9710	265,347		rPt-402480	256,854		rPt-401495	229,582
	rPt-399998	267,541		rPt-398543	257,403		rPt-507302	231,302
	rPt-390151	268,835		rPt-401486	258,249		rPt-399979	232,333
	rPt-508769	271,443		rPt-509513	259,441		rPt-505323	233,439
	rPt-506141	273,016		rPt-390337	260,076		rPt-402366	240,117
	rPt-401565	275,092		rPt-505369	261,319		rPt-400035	247,91
	tPt-5060	278,628		rPt-506030	262,078		rPt-508144	251,73
	rPt-411231	282,479		rPt-508374	263,657		rPt-401721	252,808
	rPt-2311	284,051		rPt-399597	264,455		rPt-389372	256,13
	rPt-389649	284,519		rPt-506473	264,505		rPt-506625	262,283
	rPt-508412	285,738		rPt-507838	264,516		rPt-508293	268,914
	rPt-506246	286,684		rPt-506236	264,71		rPt-505141	282,433
	rPt-401500	287,26		rPt-509154	277,649		rPt-506657	293,627
	rPt-507148	289,389		rPt-399948	279,457		rPt-411351	298,324
	wPt-7796	291,32		rPt-509728	289,142		rPt-507165	317,767
	rPt-509392	291,389		rPt-399991	289,448			
	wPt-117257	291,428		rPt-505424	289,853			
	rPt-399887	301,327		rPt-508092	293,459			
	rPt-398817	307,185		rPt-390566	294,694			
	rPt-507784	312,386		rPt-508981	296,112			
	rPt-505251	313,892		rPt-508690	298,306			
	rPt-507480	314,822		rPt-509172	300,6			
	rPt-509355	319,184		rPt-400060	303,004			
	rPt-508272	319,666		rPt-399245	303,01			
	rPt-506308	321,469		rPt-505959	303,704			
	rPt-507969	330,652		rPt-402206	304,924			
	rPt-410944	333,244		rPt-505812	305,659			
	rPt-507703	338,169		rPt-505267	306,903			
	rPt-507884	340,848		rPt-389611	307,673			
	rPt-401379	341,76		rPt-411373	307,726			
	rPt-509422	343,027		rPt-506435	307,727			
	rPt-509650	343,059		rPt-505383	307,727			
				rPt-508968	307,727			
				rPt-400800	307,885			
				rPt-390413	310,599			
				rPt-400179	315,234			
				rPt-400044	315,329			
				rPt-508512	316,086			
				rPt-401099	317,43			
				rPt-509220	321,41			
				wPt-344347	322,056			
				rPt-400037	325,203			
				rPt-507643	346,873			
				rPt-401636	349,277			
				rPt-389567	352,772			

				rPt-410775	354,141			
				rPt-402522	354,597			
				rPt-507502	355,908			
				rPt-390469	356,607			
				rPt-509502	360,183			
				rPt-401711	364,087			
				rPt-509063	364,768			
				rPt-508935	380,399			
