# Supplementary material for: Genetic architecture of complex agronomic traits examined in two testcross populations of rye (Secale cereale L.)
Source: BMC Genomics. 2012 Dec 17;13:706. doi: 10.1186/1471-2164-13-706 (PMC3566906; doi:10.1186/1471-2164-13-706)
Supplement: Additional file 4 — Quantitative trait loci of four agronomic and four quality traits in Pop-A. [file 1471-2164-13-706-S4.pdf]

Additional file 4 - Quantitative trait loci of four agronomic and four quality traits in Pop-A .

No.	Chr.	Position [cM]	Flanking marker left	Flanking marker right	Donor	Effect[a]	R^2_{adj} [b]	Frequency of occurrence[c] [%]	QTLxE[d]	Profile LOD
Grain yield [Mg ha^{-1}]										
1	1	16	c935_1675	c3264_1316	Lo90	0.054**	3.67	10.4		4.17
Plant height [cm]										
1	1	90	c16840_104	c12509_464	Lo90	-1.07**	13.45	36.1		6.14
2	2	44	c3286_1367	c29063_266	Lo115	-1.35**	20.76	70.1		4.77
3	5	0	c14666_171	c3661_432	Lo115	-0.90**	10.34	28.1		5.49
4	5	82	c8377_339	c63005_263	Lo115	-1.32**	18.00	50.5		4.34
5	7	140	c12187_156	rems2290xx	Lo90	-0.97**	10.84	83.4		5.10
1000-kernel weight [g]										
1	1	16	c935_1675	c3264_1316	Lo90	0.29**	11.09	75.6	**	10.86
2	3	112	c13282_921	c45674_298	Lo115	0.27**	9.74	50.2	**	5.50
3	5	32	c6032_341	c2288_628	Lo90	0.34**	15.95	68.6		4.27
4	6	34	c5931_480	c11417_584	Lo90	0.43**	23.89	91.3	**	18.37
5	7	86	c19209_624	c6557_853	Lo115	0.66**	40.49	87.2	**	11.76
6	7	158	rems2290xx	c10815_915	Lo115	0.21**	5.70	8.2	**	4.03
Single ear weight [g]										
1	1	50	rems2427xx	c313_2926	Lo90	0.04**	8.30	19.0		4.01
2	1	108	c2833_1992	c2246_416	Lo115	0.03**	8.87	12.6	**	5.10
Test weight [kg]										
1	1	80	c10013_747	c26456_212	Lo90	0.30**	18.22	99.9		7.74
2	2	12	c17666_682	c1976_1202	Lo115	0.32**	19.43	89.8		11.65
3	5	64	c15097_354	c8381_458	Lo115	0.26**	15.13	55.7		9.10
4	6	14	rems3155xx	c11610_397	Lo90	0.25**	13.13	75.3		9.89
5	6	82	rems2528xx	rems2154xx	Lo115	0.29**	16.34	48.9	**	4.34
6	7	106	c8227_1375	c6154_639	Lo90	0.30**	19.29	69.8		13.91

Additional file 4 – Continued

Falling number [sec.]										
1	1	32	c6468_788	c1269_382	Lo115	4.23**	10.39	19.9		3.59
2	4	154	c15845_566	c8433_714	Lo90	6.71**	23.24	98.4	**	12.13
Total pentosan [%]										
1	1	176	rems2307xx	c9442_315	Lo115	0.08**	15.32	90.7		13.73
2	2	0	c14558_855	c9328_635	Lo115	0.08**	13.03	71.5		11.67
3	3	72	c5758_1660	c16975_273	Lo90	0.07**	11.85	46.0		10.61
4	4	14	c51957_304	c27613_860	Lo115	0.05**	6.26	14.9		5.61
5	4	164	c9131_1242	c12942_952	Lo90	0.05**	6.94	25.3		6.21
6	5	60	c22500_246	rms1083xxx	Lo90	0.07**	11.14	74.7		9.98
7	6	84	rems2528xx	rems2154xx	Lo90	0.07**	11.87	66.2		10.64
Starch [%]										
1	2	6	c9328_635	c17165_808	Lo115	0.20**	13.85	79.6		6.95
2	6	94	c2794_1223	c20475_449	Lo115	0.27**	24.47	94.1		12.80

** Significant effect at P<0.01.
[a]Additive effect.
[b]Phenotypic variance explained by the detected QTL.
[c]Percentage of runs in which the QTL was detected (1000 cross-validation runs).
[d]QTL by environment interaction tested for significance (sequentially rejective Bonferroni F-test).
