# Supplementary material for: Genetic architecture of complex agronomic traits examined in two testcross populations of rye (Secale cereale L.)
Source: BMC Genomics. 2012 Dec 17;13:706. doi: 10.1186/1471-2164-13-706 (PMC3566906; doi:10.1186/1471-2164-13-706)
Supplement: Additional file 5 — Quantitative trait loci of four agronomic and six quality traits in Pop-B. [file 1471-2164-13-706-S5.pdf]

Additional file 5 - Quantitative trait loci of four agronomic and six quality traits in Pop-B.

No.	Chr.	Position [cM]	Flanking marker left	Flanking marker right	Parent	Effect[a]	R^2_{adj}	Frequency of occurrence[c] [%]	QTLxE[d]	Profile LOD
Grain yield [Mg ha^{-1}]										
1	1	46	rPt-508032	rPt-390314	Lo117	0.087**	16.86	57.1	**	7.65
2	2	144	rPt-402660	rPt-399303	Lo117	0.093**	9.88	54.3	**	4.17
3	2	210	rPt-398762	rPt-506176	Lo117	0.064**	7.47	21.9	**	5.56
4	3	14	rPt-7349	rPt-400833	Lo117	0.057**	8.61	29.1		3.91
5	4	306	rPt-506954	rPt-508519	Lo117	0.089**	16.68	54.3	**	6.25
6	5	318	rPt-505251	rPt-509355	Lo117	0.052**	6.97	65.9	**	6.32
7	7	156	rPt-508118	rPt-508571	Lo117	0.083**	15.53	45.8		10.28
Plant height [cm]										
1	1	220	rPt-507078	rPt-400034	Lo117	-1.18**	17.20	54.5	**	13.85
2	2	124	rPt-507837	rPt-505263	Lo115	-0.74**	7.00	35.8	**	3.83
3	2	168	rPt-398641	rPt-400282	Lo115	-1.04**	12.78	22.1	**	10.72
4	3	118	rPt-399732	rPt-399627	Lo115	-0.76**	9.12	30.3		4.92
5	4	310	rPt-508033	scm352xxxx	Lo117	-1.19**	16.62	66.1	**	9.54
6	5	144	wPt-344500	rPt-389787	Lo115	-1.18**	15.69	13.1	**	5.46
7	5	256	scm141xxxx	rms1115xxx	Lo115	-0.94**	8.53	15.4	**	4.89
8	5	306	rPt-399887	rPt-398817	Lo115	-1.50**	18.88	95.5	**	13.99
9	6	26	rPt-507510	rPt-507491	Lo117	-0.57**	5.52	5.6	**	3.63
1000-kernel weight [g]										
1	1	216	rPt-401334	rPt-507078	Lo115	0.38**	16.34	39.3	**	7.06
2	5	212	rPt-506729	rPt-399606	Lo117	0.53**	27.69	48.6	**	16.21
3	6	68	rPt-507486	rPt-389829	Lo117	0.56**	31.48	93.5	**	18.42
4	7	266	rPt-506625	rPt-508293	Lo115	0.42**	10.84	60.2		7.79

Additional file 5 – Continued

Single ear weight [g]									
1	1	232	rPt-509362	rPt-402055	Lo117	0.03**	10.32	36.3	+	4.72
2	3	174	rPt-402485	rPt-4378	Lo115	0.02**	6.54	14.6	*	3.71
3	7	56	rPt-508646	rms1187xxx	Lo117	0.03**	9.06	68.5		4.58
Test weight [kg]										
1	1	158	tPt-5537	scm004xxxx	Lo115	0.19**	5.74	35.2	**	9.84
2	2	86	rPt-509246	rPt-400938	Lo115	0.24**	8.54	25.7		5.46
3	2	248	rPt-399333	rPt-400854	Lo115	0.47**	22.78	48.9	**	16.06
4	3	6	rms1254xxx	rPt-402334	Lo117	0.26**	11.57	48.4	**	3.74
5	3	180	rPt-410952	scm294xxxx	Lo117	0.34**	15.84	96.0	**	6.23
6	4	308	rPt-508033	scm352xxxx	Lo115	0.28**	10.64	61.6	**	9.09
7	5	54	rPt-508587	rPt-7348	Lo115	0.33**	17.06	64.2	**	14.32
8	7	126	rPt-399849	rPt-401220	Lo117	0.27**	12.21	31.7	**	7.94
Falling number [sec.]										
1	4	76	rPt-506376	rPt-9258	Lo117	5.00**	12.95	58.2		6.21
2	6	66	rPt-507486	rPt-389829	Lo115	2.38**	5.39	15.6	**	3.87
Total pentosan [%]										
1	2	88	rPt-509246	rPt-400938	Lo117	0.07**	6.89	14.7	*	4.62
2	3	6	rms1254xxx	rPt-402334	Lo115	0.05**	5.87	52.0		4.28
3	6	44	rPt-401102	rPt-401349	Lo117	0.06**	7.01	21.8	**	6.35
4	7	28	rPt-508316	wPt-6821	Lo115	0.07**	7.43	16.9		4.44
5	7	134	rPt-390556	rPt-508956	Lo117	0.09**	13.21	51.3	**	8.91
Soluble pentosan [%]										
1	1	262	rPt-401014	rPt-411308	Lo117	0.03**	7.22	47.9		8.79
2	5	316	rPt-505251	rPt-509355	Lo115	0.03**	12.25	58.7		5.76
3	7	128	rPt-399849	rPt-401220	Lo117	0.03**	13.03	49.5	**	8.28

Additional file 5 – Continued

Protein [%]										
1	1	212	rPt-508906	rPt-3206	Lo115	0.11**	9.57	31.7	*	8.55
2	6	865	rPt-390263	rPt-505956	Lo117	0.09**	7.99	34.6	*	3.78
Starch [%]										
1	1	160	scm107xxxx	rPt-509169	Lo117	0.23**	20.16	90.7	**	14.23
2	2	168	rPt-398641	rPt-400282	Lo117	0.14**	8.64	64.8	**	6.24
3	3	4	rPt-401028	rms1254xxx	Lo117	0.21**	19.87	70.7		3.51
4	4	22	rPt-390550	rPt-398656	Lo117	0.17**	14.16	55.0		7.58
5	4	312	rPt-508033	scm352xxxx	Lo117	0.16**	10.58	91.1		5.96
6	5	52	rPt-505693	rPt-508587	Lo115	0.16**	7.80	30.6		3.62
7	5	198	rPt-509532	scm098xxxx	Lo115	0.28**	27.74	56.3		19.40
8	6	42	rPt-401102	rPt-401349	Lo115	0.19**	16.29	58.7	**	5.43
9	7	158	rPt-508571	rPt-508630	Lo117	0.19**	15.73	91.2	*	8.47

** Significant effect at P<0.01.

[a]Additive effect.

[b]Phenotypic variance explained by the detected QTL.

[c]Percentage of runs in which the QTL was detected (1000 cross-validation runs).

[d]QTL by environment interaction tested for significance (sequentially rejective Bonferroni F-test).
